# Development of Novel Pyridine-Thiazole Hybrid Molecules as Potential Anticancer Agents

**DOI:** 10.3390/molecules27196219

**Published:** 2022-09-21

**Authors:** Iryna Ivasechko, Ihor Yushyn, Piotr Roszczenko, Julia Senkiv, Nataliya Finiuk, Danylo Lesyk, Serhii Holota, Robert Czarnomysy, Olga Klyuchivska, Dmytro Khyluk, Nataliya Kashchak, Andrzej Gzella, Krzysztof Bielawski, Anna Bielawska, Rostyslav Stoika, Roman Lesyk

**Affiliations:** 1 Institute of Cell Biology of National Academy of Sciences of Ukraine, 14/16 Drahomanov Str., 79005 Lviv, Ukraine; 2 Department of Pharmaceutical, Organic and Bioorganic Chemistry, Danylo Halytsky Lviv National Medical University, Pekarska 69, 79010 Lviv, Ukraine; 3 Department of Biotechnology, Faculty of Pharmacy, Medical University of Bialystok, 15-089 Bialystok, Poland; 4 Department of Synthesis and Technology of Drugs, Faculty of Pharmacy, Medical University of Bialystok, 15-089 Bialystok, Poland; 5 Department of Organic Chemistry, Medical University of Lublin, Aleje Racławickie 1, 20-059 Lublin, Poland; 6 Department of Organic Chemistry, Poznan University of Medical Sciences, Grunwaldzka 6, 60-780 Poznan, Poland

**Keywords:** drug development, thiazoles, cancer, tumor cells, PARP

## Abstract

Novel pyridine-thiazole hybrid molecules were synthesized and subjected to physico-chemical characterization and screening of their cytotoxic action towards a panel of cell lines derived from different types of tumors (carcinomas of colon, breast, and lung, glioblastoma and leukemia), and normal human keratinocytes, for comparison. High antiproliferative activity of the 3-(2-fluorophenyl)-1-[4-methyl-2-(pyridin-2-ylamino)-thiazol-5-yl]-propenone **3** and 4-(2-{1-(2-fluorophenyl)-3-[4-methyl-2-(pyridin-2-ylamino)-thiazol-5-yl]-3-oxopropylsulfanyl}-acetylamino)-benzoic acid ethyl ester **4** was revealed. The IC_50_ of the compound **3** in HL-60 cells of the acute human promyelocytic leukemia was 0.57 µM, while in the pseudo-normal human cell lines, the IC_50_ of this compound was >50 µM, which suggests that the compounds **3** and **4** might be perspective anticancer agents. The detected selectivity of the derivatives **3** and **4** for cancer cell lines inspired us to study the mechanisms of their cytotoxic action. It was shown that preincubation of tumor cells with Fluzaparib (inhibitor of PARP1) reduced the cytotoxic activity of the derivatives **3** and **4** by more than twice. The ability of these compounds to affect DNA nativity and cause changes in nucleus morphology allows for the suggestion that the mechanism of action of the novel pyridine-thiazole derivatives might be related to inducing the genetic instability in tumor cells.

## 1. Introduction

**Figure 1:**
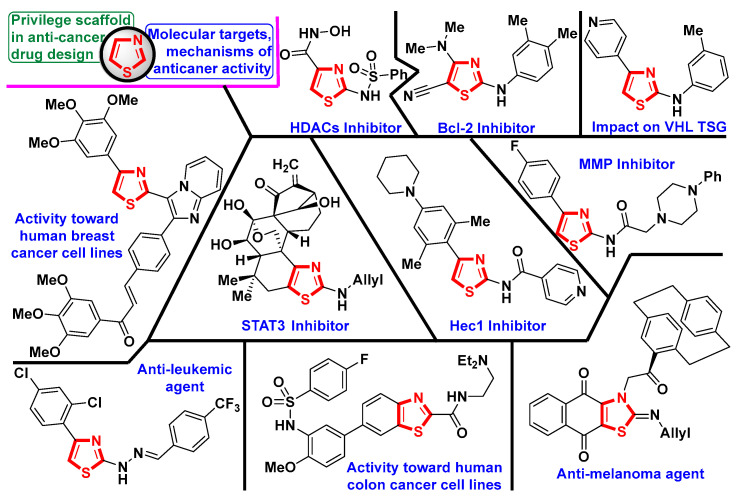
Thiazole-bearing molecules which possess anticancer activity.

Cancer is a complex disease with multiple genetic alterations including altered expression of oncogenes and tumor suppressor genes, DNA repair, tumor metabolism and other dysregulations leading to overgrowth, metastasis and drug resistance [1]. In 2021, the total number of licensed anticancer drugs counted for 270, and 243 of them were approved by the Food and Drug Administration (FDA) [2]. Searching for novel anticancer agents is an important issue in modern medicinal chemistry because current chemotherapeutics are susceptible to a common mechanism of induced drug resistance [3]. The most effective molecularly-targeted antitumor drugs, such as small molecules, monoclonal antibodies, peptides, and proteins provide a concept of selective uptake of molecules by cancer cells via interaction with specific biological targets [4]. It should be noted that small molecules are one of the most important agents for exploring pathways of suppressing cancer cell development and proliferation, as opposed to the peptides, proteins and monoclonal antibodies that require evaluation in clinical trials in order to assess their benefits [5,6]. However, the action of most small molecules is accompanied by numerous negative side effects. Moreover, the majority of the effective drugs are often financially inaccessible for a big segment of the population. Therefore, the development of anticancer molecules remains actual and is the fastest growing category in pharmacology. A better understanding of the biology of carcinogenesis may lead to the development of novel promising antineoplastic molecules, and thiazoles are among them [7,8,9]. Based on the reported research, it may be concluded that small thiazole-containing molecules utilize different mechanisms of blocking cancer cells growth via inhibition of the activity of the MMP [10], Bcl-2 [11], HDACs [12], STAT3 [13], HEC1 regulatory proteins [14], and targeting the VHL tumor suppressor gene [15] (Figure 1). 

One of the most important pathways of antitumor activity of thiazole derivatives (Figure 1) is characterized by the inhibition of protein and lipid kinases, such as c-Met kinase (anti-leukemic activity) [16], CDK1 (anti-melanoma activity) [17], CLK1 (activity against human breast cancer cell line) [18], and PI3Kα (activity against human colon cancer cell line) [19]. Due to these properties, some anticancer thiazole-bearing drugs are available on the market, namely, dasatinib (BCR-ABL kinase inhibitor) [20] and dabrafenib (B-Raf inhibitor) [21]. Other thiazole-drug candidates are under intensive preclinical/clinical investigations, specifically BCL-XL inhibitors [22], inducers of Oct3/4 [23], and dual c-Src/p38 inhibitors [24].

The aim of the present work was to design novel pyridine-thiazoles hybrid molecules and investigate their antitumor properties in accordance with a systematic study of the biological activity of thiazolidinone-related derivatives [25,26].

## 2. Results

### 2.1. Chemistry 

**Scheme 1:**
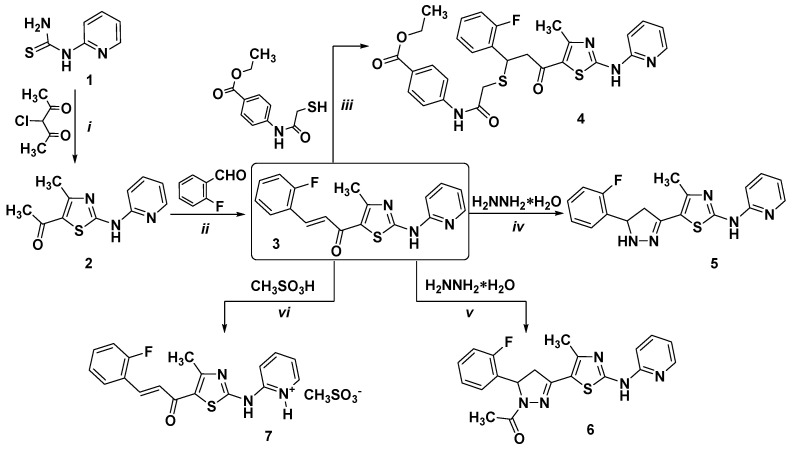
Synthesis of hybrid pyridine-thiazole derivatives. Reagents and conditions: (*i*) (pyridin-2-yl)thiourea **1** (0.01 mol), 3-chloropentane-2,4-dione (0.011 mol), AcONa (0.01 mol), AcOH (10 mL), reflux, 5 h, 74%; (*ii*) 1. comp. **2** (0.01 mol), 2-fluorobenzaldehyde (0.02 mol), *t*-BuOK (0.015 mol), EtOH (15 mL), reflux; 5 h, 2. AcOH to pH = 7.0, 80%; (*iii*) comp. **3** (0.01 mol), 4-(2-mercaptoacetylamino)-benzoic acid ethyl ester (0.010 mol), *N*-methylpiperidine (0.02 mol), EtOH (15 mL), reflux, 30 min, 81%; (*iv*) comp. **3** (0.01 mol), NH_2_NH_2_*H_2_O (0.01 mol), EtOH (15 mL), reflux, 1 h, 76%; (*v*) comp. **3** (0.01 mol), NH_2_NH_2_*H_2_O (0.01 mol), AcOH (10 mL), reflux, 1 h, 71%; (*vi*) comp. **3** (0.01 mol), CH_3_SO_3_H (0.01 mol), THF (15 mL), r.t., 1 h, 77%.

1-[4-Methyl-2-(2-pyridylamino)-thiazol-5-yl]-ethanone (**2**) was used as a key reagent for the synthesis of target derivatives (Scheme 1). For the synthesis of compound **2**, the [2+3]-cyclocondensation reaction was applied and 1-(pyridin-2-yl)thiourea **1** [27] was used as *S*,*N*-binucleophile and 2-chloroacetylacetone as the equivalent of dielectrophilic synton [C_2_]^2+^. At the next stage, the (*E*)-3-(2-fluorophenyl)-1-[4-methyl-2-(2-pyridylamino)-thiazol-5-yl]-2-propen-1-one (**3**) was synthesized from **2** and 2-fluorobenzaldehyde via the Claisen-Schmidt condensation. It is worth noting that for the mentioned reaction, the application of potassium *tert*-butylate as a catalyst in the ethanol medium led to a higher product yield and purity level, in comparison with using of potassium hydroxide (ethanol medium), as a catalyst [28]. The following synthetic design was based on the transformations of “enone” fragment of compound **3**. The Michael adduct **4** was obtained with a yield of 80% by reflux of **3** and 4-(2-mercaptoacetylamino)-benzoic acid ethyl ester with using of *N*-methylpiperidine catalyst via Michael reaction. The interaction of **3** with hydrazine hydrate [29] in ethanol medium led to pyrazoline-thiazole-pyridine hybrid **5**. Whereas using of the glacial acetic acid medium in the interaction of **3** with hydrazine hydrate led to the acetylation of the nitrogen atom in the pyrazoline ring and obtaining product **6**. Methanesulfonate **7** was synthesized from **3** and methanesulfonic acid with the aim of obtaining derivatives with improved water solubility for this type of hybrid molecules.

The structures of the synthesized compounds **3**–**7** were confirmed by the ^1^H, ^13^C NMR, and LC-MS spectra (copies of the corresponding spectra are presented in the Supplementary Material). In the ^1^H and ^13^C NMR spectra, all atoms signals are presented in the corresponding regions. In the ^1^H NMR spectra of compounds **3** and **7**, the enone fragment (CH=CHCO) appears as two doublets at 7.12–7.69 ppm with a spin-spin coupling constants of ~15.5 Hz, which indicates the *trans*-configuration of this residue and the existence of compounds in the form of *E*-isomers. The protons of the pyrazoline moiety in compounds **5** and **6** resonate with the characteristic pattern for ABX system with the relevant spin-spin coupling constants. The thiazole core carbon and aromatic carbons signals were overlapping in the ^13^C NMR spectrum of the compound **4**.

**Figure 2:**
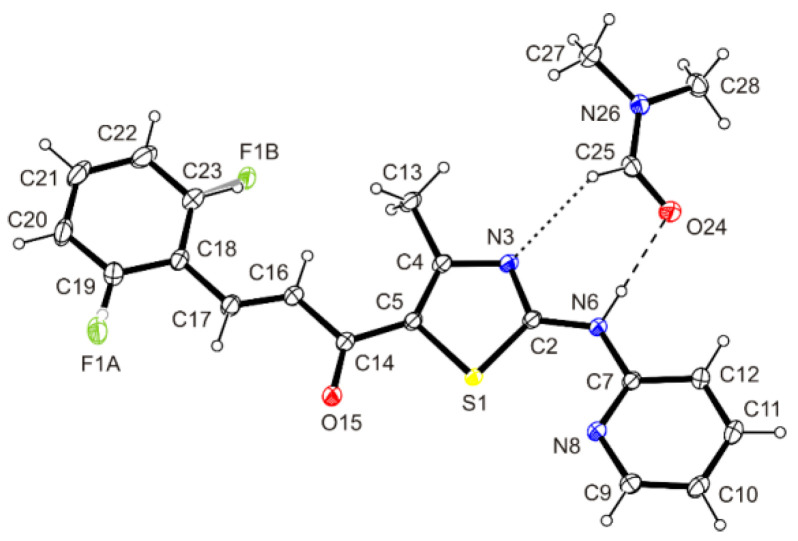
ORTEP view of 3*DMF showing the atomic labelling scheme. Non-H atoms are drawn as 30% probability displacement ellipsoids and H atoms are drawn as spheres of arbitrary size.

**Figure 3:**
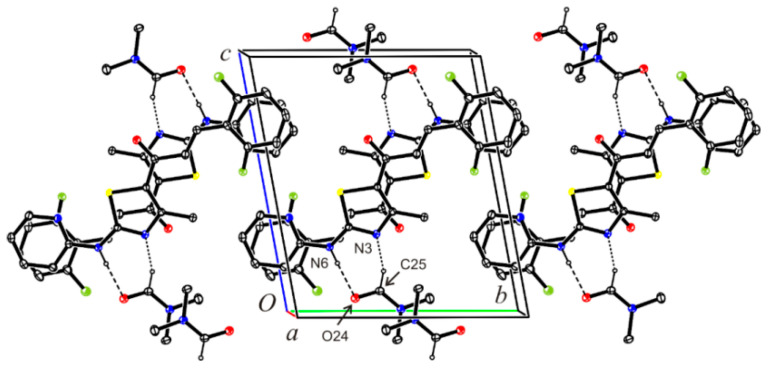
ORTEP view of the molecular packing in the crystal structure of 3*DMF.

**Figure 4:**
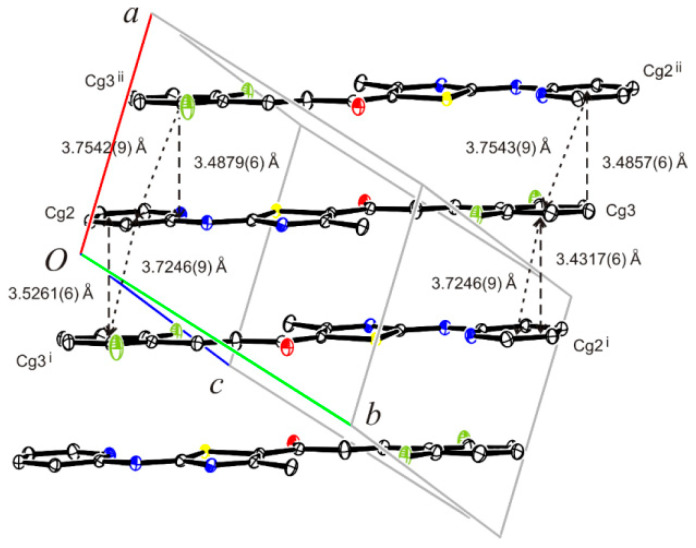
π···π Interactions between pyridine-2-yl (*Cg*2) and 2-flourophenyl (*Cg*2) systems. Hydrogen atoms have been omitted for clarity. Symmetry codes: (i) 1 − *x*, 1 − *y*, 1 − *z*, (ii) 2 − *x*, 1 − *y*, 1 − *z*.

**Figure 5:**
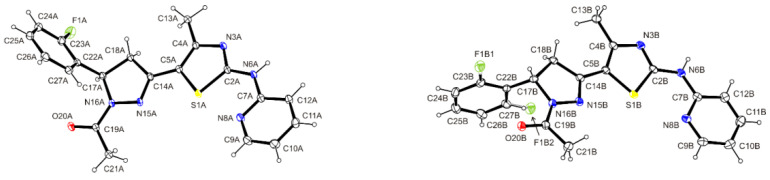
ORTEP view of the two symmetry-independent molecules of **6**, showing the atomic labeling scheme. Non-H atoms are drawn as displacement ellipsoids at the 30% probability level. H atoms are drawn as spheres of arbitrary radii.

**Figure 6:**
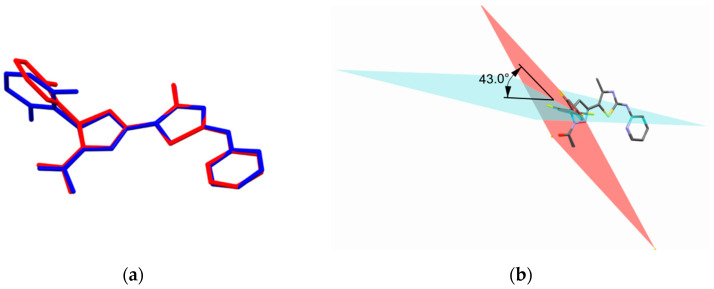
(**a**) Overlay of two symmetry-independent molecules A (red) and B (blue) by the least-squares fitting of the 4-methyl-1,3-tiazole systems; (**b**) 3D stick representation with indicated planes of phenyl ring in molecules A (pink plane) and B (blue plane).

Structural features of the synthesized derivatives were confirmed by X-ray diffraction study of compounds **3** and **6**. The crystallized compounds have the structure of (*E*)-3-(2-fluorophenyl)-1-[4-methyl-2-(2-pyridylamino)-thiazol-5-yl]-2-propen-1-one (3*DMF) and 1-{5-(2-fluorophenyl)-3-[4-methyl-2-(2-pyridylamino)-thiazol-5-yl]-4,5-dihydropyrazol-1-yl}-ethanone (**6**); the crystal of the first material is in the form of dimethylformamide solvate in a molar ratio of 1:1. In 3*DMF solute and solvent molecules are linked by hydrogen bonds (Figure 2, Figure 3 and Figure 4, Table 1). Both analyzed compounds have been crystallized in the same space group *P*$\bar{1}$, whereby the crystal of **6** has two independent molecules (A and B) in the asymmetric unit (Figure 5), which differ to a moderate degree in conformation (r.m.s.d. = 0.460 Å). The conformational differences between the molecules A and B are related to the angular rearrangement of their *o*-fluorophenyl moieties (Figure 6a). The interplanar angle found is about 43° (Figure 6b).

**Figure 7:**
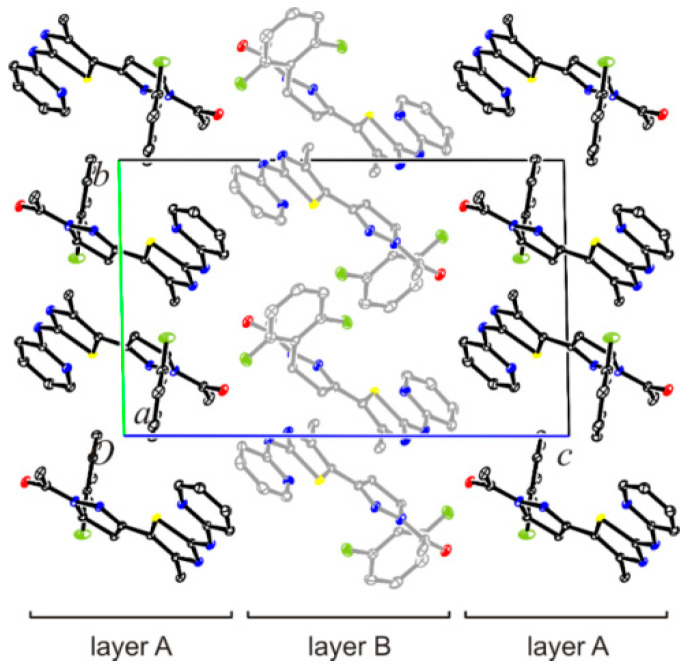
ORTEP view of the molecular packing in the crystal structure of compound **6**.

**Figure 8:**
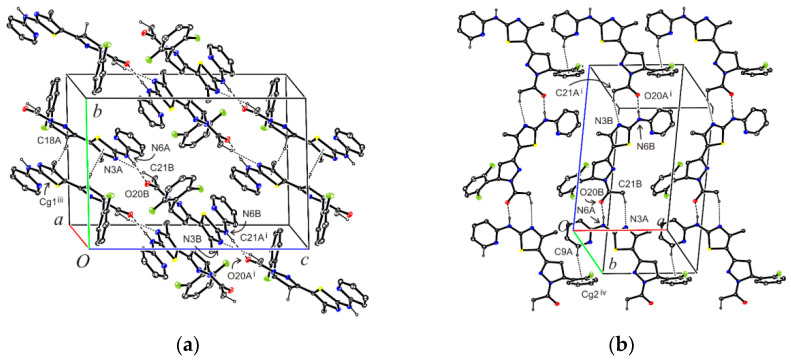
Two views of the unit cell (**a**) and (**b**) showing N―H···O and C―H···N hydrogen bonding and C―H···*Cg* intermolecular interactions in the crystal structure of **6**. The H atoms not involved in hydrogen bonds have been omitted for clarity; *Cg*1 = thiazole system, *Cg*2 = phenyl system.

X-ray analysis has shown that the molecules of two investigated compounds **3** and **6** contain an amidine group (-N=C-NH-), in which the nitrogen atom N-3 has an imine character, and the nitrogen atom N-6 is amine. In both crystals, the position of the amidine H atom was obtained from the difference Fourier map and was refined freely. Furthermore, its existence in the N6 position was confirmed by intermolecular hydrogen bonds of the N-H···O type (Table 1 and Table 2, Figure 2, Figure 7 and Figure 8) in which the N atom acts as a proton donor. The observation made for compounds **3** and **6** is rather typical for compounds containing in their molecular structure the *N*-(1,3-thiazol-2-yl)pyridin-2-amine fragment. The same tautomeric form we found in all nine related structures [refcodes: CUPLEH, GUPGAC, KIBTAS, ODAKIR, ODAKOX, OPAZUD, WIPYIF, XOVJAV, LEGWAZ] deposited in the Cambridge Structural Database, version 5.39 [30].

Structural studies have shown that the molecule of **3** is almost flat in the crystal (r.m.s.d. = 0.0474 Å). Simultaneously, the 2-pyridylamino group within the 4-methyl-2-(pyridin-2-ylamino)-1,3-thiazol-5-yl moiety adopts synperiplanar conformation with respect to the S1–C2 bond belonging to the 4-methyl-1,3-thiazole system. This arrangement is confirmed by the torsion angle S1-C2-N6-C7, 2.71(19). The positioning of the 3-(2-fluorophenyl)prop-2-enoyl moiety in the molecule is described with torsion angles C4-C5-C14-O15, O15-C14-C16-C17 and C14-C16-C17-C18 [177.59(13), −3.3(2) and −179.35(13)°, respectively]. The first two show that the C4-C5/C14-O15 double bond pair has the *s*-trans conformation and the C14-O15/C16-C17 double bond pair has the *s*-cis conformation. The third mentioned torsion angle indicates the trans configuration of the C14-C16/C17-C18 bond pair. The C16-C17 bond length, 1.309(2) Å, found in the prop-2-enoyl framework, confirms the presence of a double bond between the mentioned atoms (literature C-C double bond length is 1.331(1) Å [31]).

In both independent molecules A and B of **6**, the thiazole and pyridine systems within the planar 2-(2-pyridinylamino)-thiazole moiety are positioned synperiplanar, as in the molecule of **3**. The S1-C2-N6-C7 torsion angle for the molecules A and B is −5.5(2)° and 2.4(2)°, respectively. Within the 1-acetyl-5-(2-fluorophenyl)-4,5-dihydro-1*H*-pyrazol-3-yl fragment, a flat pyrazoline ring forms with the acetyl group and the 2-fluorophenyl system dihedral angles of 7.3(2)° and 67.94 (4)° (molecule A) and 5.7(3)° and 84.71(4)° (molecule B). Figure 3 shows the molecular packing in the unit cell of 3*DMF. In the crystal lattice, almost flat solute molecules are arranged in piles growing along the axis a. Within a pile, the contacts *Cg*2···*Cg*3^i^, *Cg*2···*Cg*3^ii^ (symmetry codes: (i) 1 − x, 1 − *y*, 1 − z, (ii) 2 − *x*, 1 − *y*, 1 − *z*; *Cg*2: 2-pyridinyl, *Cg*3: 2-fluorophenyl systems) between the molecules are also observed (Figure 4).

In the crystal lattice of **6**, layers parallel to the ab plane are formed separately by molecules A and B, and B molecules are observed (Figure 7). The molecules A and B from neighboring layers are linked by N6A-H6A···O20B, N6B0H6B···O20A^i^, C21B-H21B···N3A and C21A-H21E···N3B^ii^ hydrogen bonds into chains growing along the [011] direction. Apart from hydrogen bonds in the crystal, the interactions of C9A―H9A···*Cg*1^iii^ and C18-H18C···*Cg*2^iv^ are also observed. The interactions connect chains of molecules to form the layers that grow parallel to the (011) plane.

### 2.2. Biological Evaluations

#### 2.2.1. In Vitro Study of the Anticancer Activity in 60 Lines under the NCI DTP Screening

For a preliminary assessment of the antitumor activity of pyridine-thiazoles, a screening study of the compounds **4**, **5** and **6** was conducted within the Developmental Therapeutic Program (DTP) by the National Cancer Institute (NCI, Bethesda, Rockville, MD, USA). Primary anticancer assays (Table 3) on a panel of 60 cancer cell lines at one dose-assay (10^−5^ M) were performed according to the NCI protocol, as described elsewhere [31,32,33,34,35]. The most active anticancer agent turned out to be compound **4**. It inhibited the growth of all 60 tested cancer cell lines by more than 50%, with the range of growth −76.78–41.12%. Moreover, this compound showed not only a cytostatic effect, but also cytotoxic properties causing the death of cancer cells. Non-small cell lung cancer (NCI-H460, NCI-H522), melanoma (LOX IMVI, SK Mel-2), colon (Colo 205, HCT-15, HT29, KM12) and CNS (SF-539, U251) cancer cell lines were most sensitive to the action of compound **4**. 

Compared to the compound **4,** parazoline-pyridine-thiazole hybrid molecules **5** and **6** showed moderate antitumor activity with a mean growth percent of about 50%. Therefore, the additional introduction of a pyrazoline fragment into the structure of the studied compounds leads to a certain loss of antitumor effect, which, nevertheless, is potentially interesting for the design of new anticancer molecules. Thus, the leukemic cells of CCRF-CEM, HL-60(TB), K-562, and SR lines, lung cancer cells of NCI-H522 line, melanoma MDA-MB-435 line, as well as breast cancer MDA-MB-468 line were the most sensitive to the action of the compound **5**. For compound **6**, a selectivity of antitumor action towards leukemia (K-562, SR), melanoma (SK Mel-5), and breast cancer (T-47D) cell lines was also observed.

Taking into account a significant inhibition of the viability of tumor cells by **4**, it was selected for in-depth screening of its action towards a full panel of cells in a broad range of concentrations [31,32,33,34,35]. The compound **4** inhibited the viability of all tested cancer cell lines at the micromolar concentrations (Table 4). The average meaning of the dose-response parameter GI_50_ (molar concentration of the compound that inhibits 50% net cell growth) was 31.7 µM. The TGI (molar concentration of the compound leading to the total inhibition) and LC_50_ (molar concentration of the compound leading to 50% net cell death) were mainly >100 µM. It is important to note that the compound **4** was active in the concentration range of 2.52–8.29 μM towards the following cell lines: RPMI-8226 (leukemia); NCI-H226 (non-small cell lung cancer); SNB-75, SF 539 (CNS cancer); M14, MDA-MB-435 (melanoma); UO-31, RXF 393 (renal cancer); MDA-MB-468 (breast cancer). The studied compound showed the highest efficiency against the melanoma line LOX IMVI, because the cytotoxicity was at the submicromolar level (0.312 μM).

The selectivity indices of the compound **4** were calculated by dividing the full panel MG_MID GI_50_ (μM) of the **4** by the individual parameter’s value for each cell line (μM). Ratios were between 3 and 6 mean moderate selectivity; ratios greater than 6 indicate high selectivity toward the corresponding cell line, while the compounds not addressing any of these criteria were rated nonselective [36]. At the GI_50_ level, the derivative **4** did not show a selectivity of action against certain types of cancer (SI = 0.61–1.41). At the same time, regarding the individual cell lines, the best SI values were observed for such lines: RXF 393 (renal cancer, SI = 12.2) and LOX IMVI (melanoma, SI = 101.6).

#### 2.2.2. Application of MTT Assay for Measurement of Viability of HCT-116, HCT-116 p53 (−/−), MCF-7, Jurkat, HL-60, A549, SK-OV-3, and KB3-1 Cell Lines

Taking into account the results of the preliminary assessment of the antitumor potential of pyridine-thiazoles, synthesized compounds were studied for their antineoplastic activity on a panel of cancer cell lines (Table 5) including colon (HCT-116, HCT-116 p53 (−/−), breast (MCF-7), leukemia (Jurkat, HL-60), lung (A549), ovarian (SK-OV-3), cervix (KB3-1). The compounds **3** and **4** demonstrated similar cytotoxic effect toward tumor cells, IC_50_ ranged from 0.57 µM and to 7.8 µM depending on tumor cell line. Derivative **7** acted more weakly compared with **3** and **4**. These compounds possessed low toxicity (IC_50_ > 50 µM) towards pseudo-normal cell lines (human embryonic kidney HEK293 cells, and human epidermal keratinocytes of HaCaT line), but murine macrophages of J774.2 line and BALB-3T3 (normal mouse fibroblasts) were more sensitive, IC_50_ ranged from 7.18 to 34.16 µM. Compounds **1** and **2**, the precursors of **3**, **4**, and **7**, demonstrated a lower cytotoxic effect towards tumor cells, and the IC_50_ was higher than 50 µM. The IC_50_ of **5** and **6** ranged from 30 µM to >50 µM. 

Based on the obtained data, compounds **3** and **4** had strong and similar cytotoxic activity. It should be noted that these compounds acted much more specifically towards tumor cell lines and were less harmful for the normal cells compared with doxorubicin used as the positive control. In the case of SK-OV-3 cells, the derivative **4** was more active, and IC_50_ was 7.87 µM compared with IC_50_ > 50 of **3**. Another two compounds, **5** and **6**, which differ from the compounds **3** and **4** by the presence of a pyrazoline moiety, showed a significantly lower activity.

**Figure 9:**
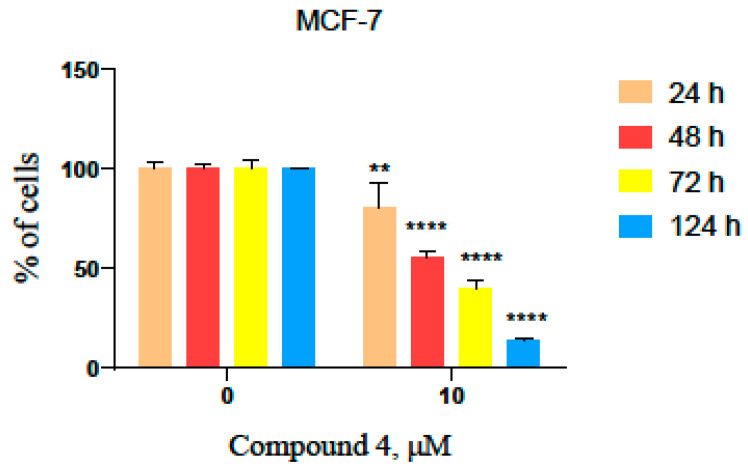
Time-dependent activity of **4** at concentration 10 µM after 24, 48, 72 and 124 h of treatment, MTT assay (** *p*  <  0.01, **** *p*  <  0.0001 compared with control).

The time-dependent cell viability data were obtained as a result of 24, 48, 72, and 124 h MTT tests performed with the most active compound **4** on MCF-7 cell line. A strong correlation was detected between the treatment time and an anti-proliferative potential. The cytotoxic effect of this compound develops after 48 h of incubation. This feature may explain the difference of cell viability compared with the NCI results which were obtained at 48^th^ hour of incubation of cells with derivative **4**. A new pyridine-thiazole derivative was found to kill only 19.9% of the growing cells at the 24th hour, while at the end of 124 h, 90% of the breast cancer cells died, as seen in Figure 9. The result obtained at 48^th^ hour of treatment of the MCF-7 cells with the compound **4** in concentration 10 µM correlates to some extent with data of the NCI screening. At that time point, 43% of the cells died. 

#### 2.2.3. Application of Clonogenic Assay for Measurement of Single Cell Growth Inhibition

**Figure 10:**
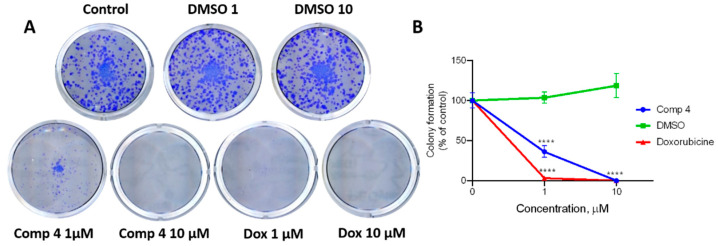
Clonogenic survival, performed by incubating of the MCF-7 cells with the compound **4** for 10 days. (**A**) Representative cell culture wells showing results from a clonogenic survival assay performed with cells treated with **4**, DMSO, and Doxorubicin in indicated concentrations. (**B**) This graph represents a loss of clonogenic survival of MCF-7 cells under the above-mentioned conditions (**** *p*  <  0.0001 compared with control).

This assay essentially tests every cell in the population for its ability to undergo “unlimited” division [37]. New pyridine-thiazole hybrid molecules **4** had a strong influence on colony formation. At 1 µM concentration, it reduced the ability of the MCF-7 single cell to grow into a colony to 36%. Doxorubicin, which was used as a positive control, showed significantly higher such ability (3% compared with control) (Figure 10A). No colony formation was observed upon 10 µM of **4** treatment during 10 days, as shown in (Figure 10B). With a concentration equivalent to 10 µM of the compound **4**, the DMSO increased the cell’s ability to grow in colony.

#### 2.2.4. Application of Soft Agar Assay for Evaluation of the Inhibition of Tumorigenicity In Vitro

**Figure 11:**
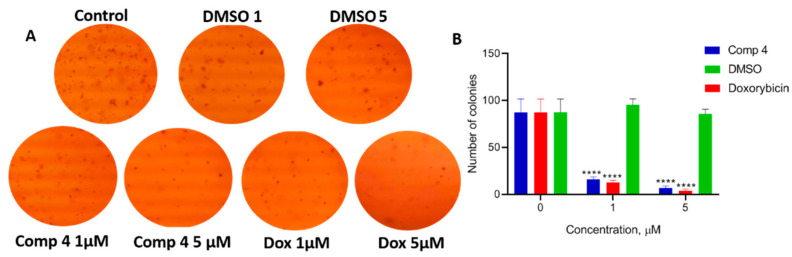
Compound **4** reduces the size and number of colonies of MCF-7 cells. Results of soft agar drug sensitivity assay (day 12). (**A**) Representative cell culture wells. (**B**) Graphical data (**** *p*  <  0.0001 compared with control).

It was proposed that testing of drugs in a 3-dimensional (3D) format, such as soft agar, is more similar to the in vivo cellular microenvironment and the results of such testing have a good correlation with in vivo conditions [38]. In the case of incubation of MCF-7 breast cancer cells with the studied compound, a marked dose-dependent suppression of the colony formation was observed (Figure 11B). The size of colonies was significantly smaller (Figure 11A), most of the colonies were ˂50 µm in size, and their number was significantly lower compared with control and DMSO treatment, but these colonies were larger in size compared with the effect of doxorubicin used as a positive control.

The results of colony formation assay demonstrated that the compound **4** inhibited the viability of MCF-7 cells in a dose-dependent manner. These results are in agreement with the data of soft agar assay. A major advantage of this technique is that the semi-solid matrix selectively favors the viability of cells that can proliferate in an anchorage-independent manner [39,40]. Altogether, these data indicated that the derivative **4** exhibited a significant anticancer activity against breast cancer cells. 

#### 2.2.5. Cytomorphological Changes Induced by Novel Hybrid Pyridine-Thiazole Derivatives

**Figure 12:**
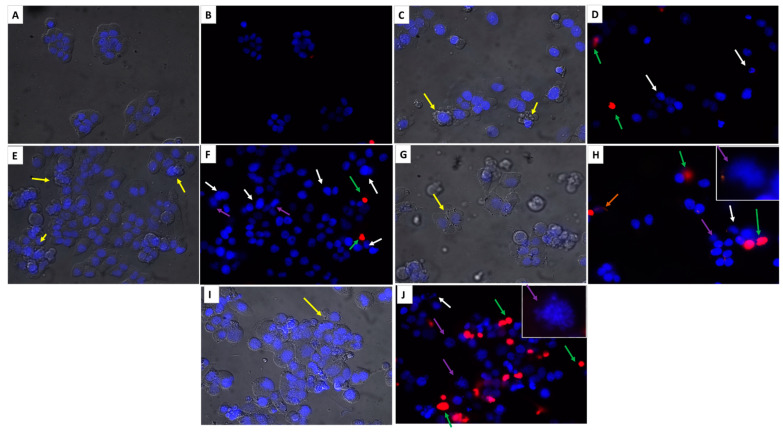
Fluorescent images of morphological changes in KB3-1 cells: non-treated cells, control (**A**,**B**); doxorubicin, 1 µM (**C**,**D**); cisplatin, 5 µM (**E**,**F**); compound **3**, 5 µM (**G**,**H**); compound **4**, 5 µM (**I**,**J**). Exposure time—48 h. Cells were stained with Hoechst 33342 and Propidium Iodide fluorescent dyes. The yellow arrow indicates cells with membrane blebbing, white—condensed chromatin, green—dead cells, purple—mitotic catastrophe, and red—multinucleated cells. Magnification ×400.

Next, we investigated morphological changes in KB-3-1 cells under the action of two of the most active compounds **3** and **4**. The majority of cells treated with the compound **4** exhibited changes in nuclear morphology, similar to a mitotic catastrophe (Figure 12I,J). In addition, we observed multinucleated giant cells. Thus, compound **4** might induce genetic instability in the treated cells resulted in such morphological changes. The derivative **3**, the precursor of **4** (Figure 12G,H), and cisplatin (Figure 12E,F), which was used as a positive control, caused significantly less DNA damage in KB3-1 treated cells, although cells with the mitotic catastrophe were also observed. In contrast, doxorubicin (also used as a positive control, Figure 12C,D) induced cell membrane damage and membrane blebbing to a greater extent compared to the effect of the compounds **3** and **4**. The condensed chromatin, a characteristic feature of apoptosis, and dead cells were observed under the action of all studied compounds. Non-treated cells in the control (Figure 12A,B) had a morphology characterized for KB3-1 cell line.

**Figure 13:**
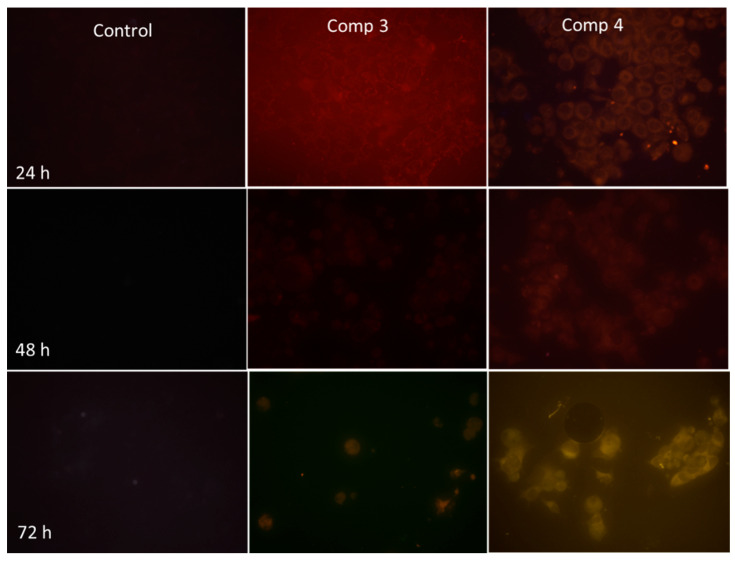
Red-yellow fluorescence of compounds **3** and **4** observed in the MCF-7 cells. Cells were incubated with pyridine-thiazole derivatives at the concentration of 5 µM for 24, 48, and 72 h. Magnification ×400.

The status of cell morphology is an important indicator for monitoring cellular response to treatment and for analyzing the mechanisms of compound action. We used two different positive controls, doxorubicin and cisplatin, in order to compare the similarity of changes in morphology under the action of studied derivatives. In all studied cases, the morphological changes in the KB-3-1 cells were similar to those caused by cisplatin. Additionally, we examined the ability of studied compounds to fluorescence in the treated cells (Figure 13). The compounds **3** and **4** demonstrated the red-yellow fluorescence in the MCF-7 cells. Thus, it can be assumed that the compounds penetrate into the cells and the derivative **4** was accumulated in a time-dependent manner close to the nucleus of the cell. 

#### 2.2.6. DNA Binding by Novel Hybrid Pyridine-Thiazole Derivatives

Since the studied compounds caused morphological changes in the nucleus, we decided to study the opportunity of the novel hybrid pyridine-thiazole derivatives to act on the DNA. The oxidative reaction of KMnO_4_ was used to study the selective oxidation of pyrimidine bases, particularly thymidine, in the mismatched or single-stranded regions of DNA with potassium permanganate. The compounds which interact with DNA distort its duplex structure, thus, subjecting pyrimidine bases to the oxidation by KMnO_4_, generated products can be detected using UV/VIS spectrophotometry. Thus, it is possible to identify different classes of DNA-binding compounds (intercalators, binding grooves, and alkylating agents) [41].

**Figure 14:**
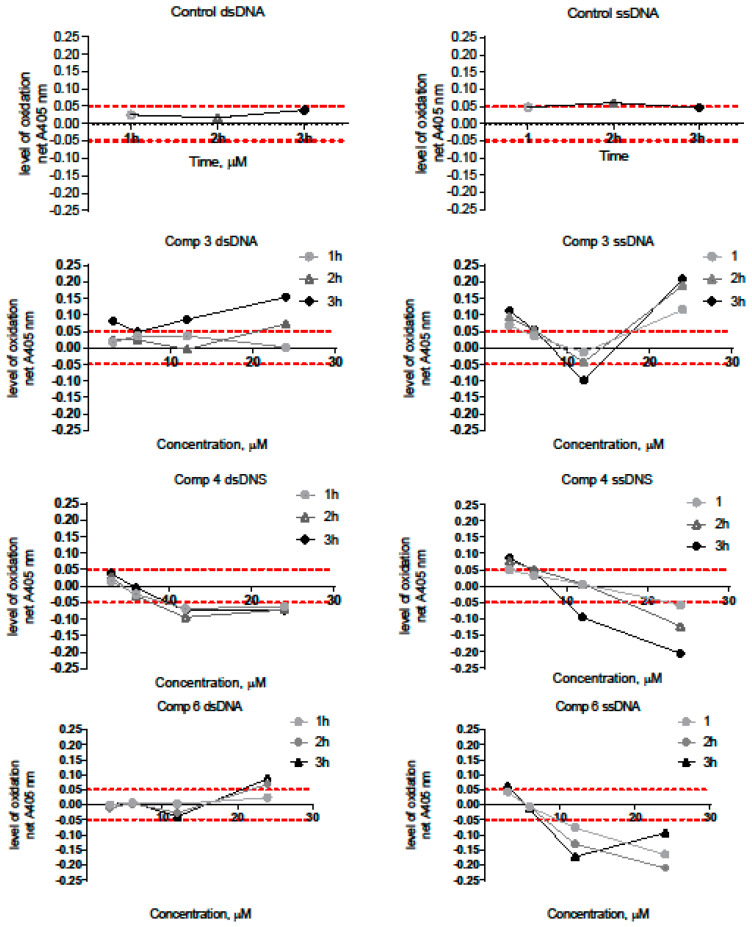
DNA damage induced by the compounds during various periods of incubation. DNA-binding compounds were defined as falling within the groups where the net change in absorbance between zero and various time points was >0.05 or <−0.05 (red line).

The results of the conducted studies indicate that the level of permanganate oxidation of salmon sperm DNA induced by new compounds demonstrate a similar tendency and a stronger interaction was identified with single-strand DNA (ssDNA), than with double-strand DNA (dsDNA) (Figure 14). All studied derivatives showed dose and time-dependent effects. A NetAbs for reaction with ssDNA was from −0.098 to 0.209 for compound **3**, from 0.206 to 0.086—for **4**, and from −0.214 to 0.082—for **5**. Comparing the effect of compounds on dsDNA, it can be noted that compound **3** showed the strongest influence on dsDNA compared with **4** and **5**. The net change in absorbance between zero and various time points ranged from −0.004 to 0.154 (**3**), and −0.094 to 0.04 (**4**). The compound **5** interacted with dsDNA to the least extent, NetAbs was from −0.038 to 0.088.

#### 2.2.7. Novel Pyridine-Thiazole Hybrid Molecules Induce Apoptosis by Decreasing Mitochondrial Membrane Potential

**Figure 15:**
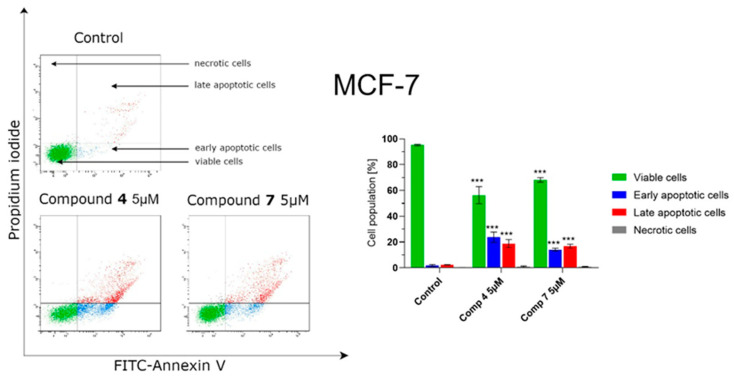
Flow cytometry analysis of apoptosis induction in the MCF-7 breast cancer cells after 24 h incubation with the compound **4** (5 µM) and the compound **7** (5 µM) staining with Annexin V-FITC/PI). The obtained results are presented as mean values ± SD obtained from three independent experiments (n = 3) conducted in duplicate. *** *p* < 0.001 compared with. control.

Apoptosis is a natural programmed mechanism required to maintain homeostasis in the body, and in the case of cancer cells, it is an effective and essential process leading to their death and elimination [42]. We evaluated the induction of apoptosis by means of flow cytometry after 24 h of incubation of MCF-7 breast cancer cells with the compounds **4** and **7** (both 5 µM). Annexin V-FITC and Propidium Iodide were used for apoptosis measurement. The compound **4** and **7** caused a significant increase in number of the apoptotic cells (sum of early and late apoptotic cells) in the MCF-7 cells: 42.7 ± 7.0% of apoptotic cells were detected for compound **4**, and 31.0 ± 1.9%—for compound **7** (Figure 15). The amount of the apoptotic cells was significantly increased compared with the control (4.4 ± 0.6%).

**Figure 16:**
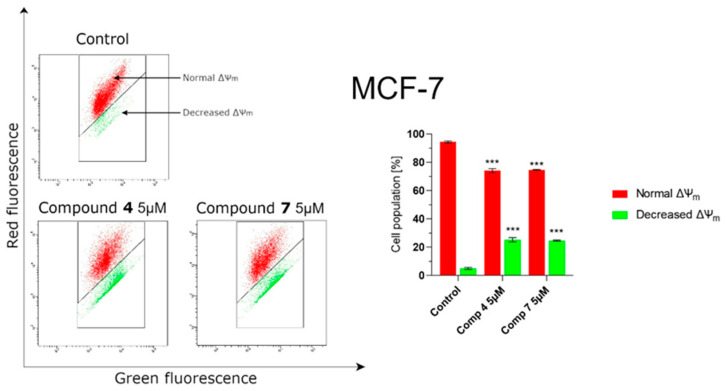
Flow cytometry analysis of mitochondrial membrane potential changes (ΔΨ_m_) in the MCF-7 breast cancer cells after 24 h incubation with the compound **4** (5 µM) and compound **7** (5 µM). The obtained results are presented as mean values ± SD obtained from three independent experiments (n = 3) conducted in duplicate. *** *p* < 0.001 compared with control.

A decrease in the mitochondrial membrane potential (MMP, ΔΨm) is one of the earliest changes observed at apoptosis [42]. Apoptosis, which proceeds through the mitochondrial pathway, demonstrates an increase in the permeability of the internal and external mitochondrial membrane, which is associated with changes in the trans-membrane mitochondrial potential [43]. We used a flow cytometer and the cationic dye JC-1 to determine changes in ΔΨm of breast cancer cells (MCF-7 line) treated for 24 h with the compound **4** and **7** at a concentration of 5 µM. Both tested compounds demonstrated significant effect on the ΔΨm (Figure 16). The MCF-7 cells had depolarized mitochondria, reaching 25.3 ± 1.5% and 24.7 ± 0.4% of the cell population treated with the compound **4** and **7**, respectively. In the control, there were only 5.1 ± 0.7% cells with a decreased ΔΨm. These findings suggest that apoptosis induced by the compound **4** and **7** may follow an intrinsic pathway, as manifested by a reduction in the ΔΨm.

#### 2.2.8. PARP1 Inhibition in Treated Tumor Cells

It is known that thiazole-containing compounds were developed as potent poly ADP-ribose polymerase (PARP) inhibitors (PARPi) [44,45]. Thus, 2-aminothiazole derivatives are of special interest as PARPi [46]. The enzymes involved in the DNA repair are the main targets of agents called the PARPi. Such molecules stop this process, thus, contributing to the transformation of single-strand breaks into double-strand ones. It was found that PARP1 inhibitors block the same location in the catalytic center and may have a diverse clinical effectiveness. PARPi molecules can disrupt PARP1 allostery in two ways: (1) drive a release of PARP-1 from the DNA, and (2) promote the retention. These insights helped us to construct the most effective PARPi which traps PARP at the site of the DNA break, generating a lesion that leads to cytotoxicity, especially in tumor cells with deficiencies in the repair of the DNA strand breaks [47]. The application of these drugs together with other DNA repair inhibitors may help to overcome drug resistance and, thus, play an important role in the development of concept of the synthetic lethality.

**Figure 17:**
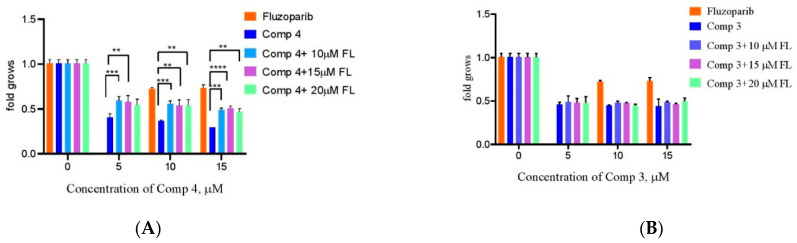
Preincubation of MCF-7 cells with PARP1 inhibitor Fluzoparib decreased their sensitivity to the treatment with compounds **4** (**A**) and **3** (**B**). MTT data on 72 h of cells incubation with studied compounds **3** and **4** (** *p*  <  0.01, *** *p*  <  0.001, **** *p*  <  0.0001).

Taking into account the above data, PARP1 inhibition in vitro was investigated using pretreatment of the MCF-7 cells with the Fluzoparib, a potent PARP1 inhibitor in the non-toxic concentrations of 5, 10, and 15 µM. It had no significant growth inhibitory effect in such doses, and a reduction of cell viability by only 10–12% was observed compared to control (non-treated) cells. The IC_50_ of the Fluzoparib was 47 µM. The preincubation step was used to bind PARP1 molecules, which were the potential targets of the studied compounds and to test if it would affect the activity of **3** and **4**. It was found that the preincubation of the MCF-7 cells with PARP1 inhibitor reduced in more than 3 times their sensitivity to **4**. The IC_50_ of this compound was 3.53 µM and it was increased to 13.95 µM in the case of cell preincubation with the Fluzoparib (10 µM) (Figure 17A). The preincubation of cells with the Fluzoparib decreased their sensitivity to the compound **3** treatment in a less pronounced manner, compared with the effect of the compound **4**. The IC_50_ of free **3** was 3.9 µM and 6.86 µM—under preincubation (Figure 17B). Since the preincubation of the MCF-7 cells with PARP1 inhibitor statistically reduced the sensitivity of these cells to the compound **4**, one can assume that this compound is a potent PARP1 inhibitor. Changes in structure of the started compound **3** may cause an enhancement of the ability of molecule **4** to inhibit the activity of PARP1.

**Figure 18:**
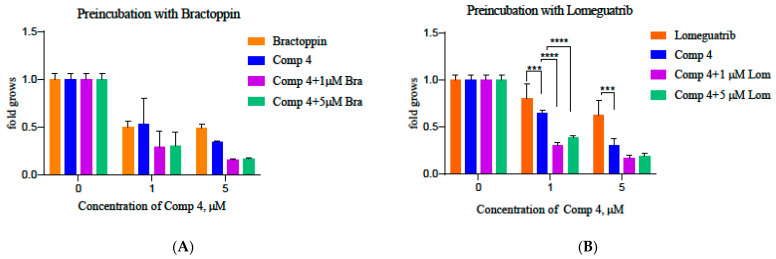
Chemical synthetic lethal interactions between PARP1 inhibition by the compound **4**, and BRCA1 (**A**), MGMT (**B**) inhibition. MTT data, 72 h of incubation (*** *p*  <  0.001, **** *p*  <  0.0001).

According to the theory of synthetic lethality, the base excision repair protein PARP1 is critical for the compensation of a loss of the activity of other repair proteins, such as BRCA1/2 (BReast CAncer gene 1/2) [48]. Here, we used MGMT (O-6-Methylguanine-DNA Methyltransferase) and BRCA1 inhibitors to block DNA repair compensatory mechanisms and to study the potential chemical synthetic lethality action of the compound **4** and these inhibitors (Figure 18). The preincubation of the MCF-7 cells with the Lomeguatrib enhanced their sensitivity to treatment with the compound **4** by 3 times, while the IC_50_ of **4**, a potent PARP inhibitor, was 2.28 µM and 0.72 µM with the MGMT inhibitor (Figure 18B). A similar tendency was obtained using Bractoppin, but without a statistical significance. In the case of BRCA1 inhibition, the IC_50_ was 0.70 µM (Figure 18A).

It was found that a combination of the compound **4** with the MGMT inhibitor showed the synergistic activity towards the MCF-7 cells, compared with their individual treatments. This feature may be interesting for further study of the action of this compound on the MGMT mutant cells, in particular, together with the DNA-binding drugs. Nowadays, a search for effective molecules and drug combinations are widely used in chemotherapy. A new combination, targeting different pathways, can reduce or delay the development of cancer resistance, enhance the sensitivity of cancer cells to anticancer drugs, and reduce toxicity by reducing the required dose of a single drug [49].

#### 2.2.9. Molecular Docking Simulations

In order to explore possible anticancer pharmacodynamics profile, docking investigation have been performed. PolyADP-ribose polymerases PARPs (PARP1 [50] and PARP2 [51]) were selected for in silico simulations. The highest binding energies and inhibition constants Ki demonstrated compound **4** to both PARPs enzymes (Table 6).

In addition, docking scores are higher, compared to the new drug Talazoparib (Talzenna by Pfizer), which allows suggesting about the potent drug-like profile of the compound **4**. In spite of good results of the biological assays, the compound **3** showed the worst binding energies to both PARPs. This fact allows the purposing of another main cytotoxic mechanism for the compound **3** or affinity to other members of PARPs family.

**Figure 19:**
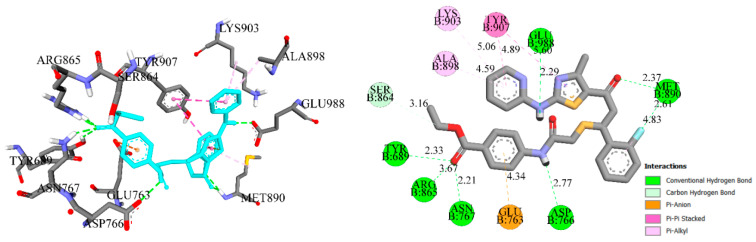
The binding of compound **4** best-generated conformer with the PARP1 (PDB 7KK4).

Compound **4** binds the side active site of the PARP1 by the extensive hydrogen-bonding and different types of Pi interactions. Additionally, the molecule forms the weak carbon-hydrogen bonds with Ser864 (Figure 19).

**Figure 20:**
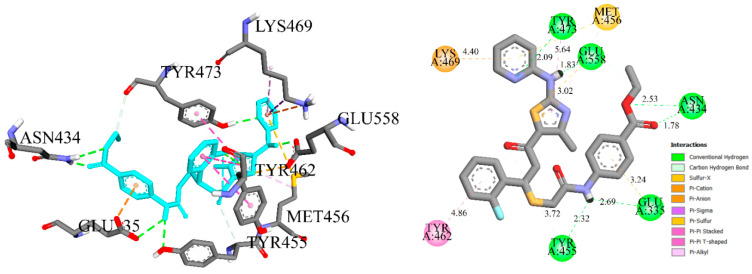
The binding of compound **4** best-generated conformer with the PARP2 (PDB 4PJV).

The compound **4** forms a complex with the PARP2 in the same way, forming 5 hydrogen bonds with the Tyr473, Glu558, ASN434, Glu335 and Tyr455 (Figure 20). In addition, all the aromatic rings of the molecule form different types of hydrophobic interactions with the same amino acids and Lys469 and Met456.

It has to be noted that interaction of the compound **4** with the PARPs differs from the other PARP inhibitors. The molecule does not occupy benzamide binding pockets of the PARPs (Gly863 and Ser904 in PARP1) and (Gly429 and Ser470 in PARP2) [51]. Additionally, the compound **4** structure is not in line with the standard skeleton of the PARP inhibitors, which consist of three parts: benzamide pharmacophore, aromatic linker and secondary or aromatic amine moiety [52]. Docking simulations and biological assays which allow suggestions about the compound **4** as a new type of PARPs inhibitor, which needs to be confirmed in further investigations. 

## 3. Materials and Methods

### 3.1. General Information

All reagents and solvents were purchased from commercial suppliers and were used directly without further purification. Melting points were measured in open capillary tubes on a BÜCHI B-545 melting point apparatus (BÜCHI Labortechnik AG, Flawil, Switzerland), and were uncorrected. The elemental analyses (C, H, N) were performed using the Perkin-Elmer 2400 CHN analyzer (PerkinElmer, Waltham, MA, USA) and were within ±0.4% of the theoretical values. The 500 MHz ^1^H and 100 MHz ^13^C NMR spectra were recorded on a Varian Unity Plus 500 (500 MHz) spectrometer (Varian Inc., Paulo Alto, CA, USA). All spectra were recorded at room temperature, except where indicated otherwise, and were referenced internally to solvent reference frequencies. Chemical shifts (δ) are quoted in ppm and coupling constants (*J*) are reported in Hz. LC-MS spectra were obtained on a Finnigan MAT INCOS-50 (Thermo Finnigan LLC, San Jose, CA, USA). The reaction mixture was monitored by thin layer chromatography (TLC) using commercial glass-backed TLC plates (Merck Kieselgel 60 F254). IR spectra were recorded with a PerkinElmer FT-IR spectrometer Spectrum Two (PerkinElmer, Waltham, MA, USA) with the universal ATR sampling. Solvents and reagents that are commercially available were used without further purification. The (pyridin-2-yl)thiourea **1** was prepared according to the method described in [27].

The cell lines used in our work were kindly provided by a Collection at the Institute of Molecular Biology and Genetics, National Academy of Sciences of Ukraine (Kyiv, Ukraine). The p53-deficient HCT-116 p53 (−/−) colon cancer cells were donated by a Collection of the Institute for Cancer Research at Vienna Medical University (Vienna, Austria). Cells were maintained in Dulbecco’s Modified Eagle Medium (DMEM, Biowest, France) or RPMI-1640 medium (Biowest, France), containing 10% of fetal bovine serum (FBS, Biowest, France) according to recommendations of American Type Culture Collection (ATCC), under the incubation conditions of 5% CO_2_ humidity at 37 °C [53].

### 3.2. Synthesis of 1-[4-Methyl-2-(2-pyridylamino)thiazol-5-yl]ethanone (***2***)

A mixture of (pyridin-2-yl)thiourea **1** (10 mmol) with the 3-chloropentane-2,4-dione (11 mmol) and anhydrous sodium acetate (10 mmol) was refluxed for 5 h in glacial acetic acid (10 mL) (monitored by TLC). Obtained solid product was collected after cooling by filtration and recrystallized from acetic acid. Yield: 74%, mp 231–233 °C. ^1^H NMR (400 MHz, DMSO-*d_6_*): δ (ppm) 2.45 (s, 3H, CH_3_), 2.56 (s, 3H, CH_3_CO), 7.01 (t, 1H, *J* = 6.1 Hz, pyridine), 7.09 (d, 1H, *J* = 8.1 Hz, pyridine), 7.75 (t, 1H, *J* = 8.1 Hz, pyridine), 8.38 (d, 1H, *J* = 4.4 Hz, pyridine), 11.76 (s, 1H, NH). LCMS (ESI): *m*/*z* 234.0 (100.00%, [M+H]^+^). Anal. Calc. for C_11_H_11_N_3_OS: C 56.63%; H 4.75%; N 18.01%. Found: C 56.50%; H 4.70%; N 18.20%.

### 3.3. Synthesis of (E)-3-(2-Fluorophenyl)-1-[4-methyl-2-(2-pyridylamino)-thiazol-5-yl]-2-propen-1-one (***3***)

A mixture of compound **2** (10 mmol) with the 2-fluorobenzaldehyde (20 mmol) and potassium *tert*-butylate (15 mmol) in ethanol (15 mL) was heated under reflux for 5 h (monitored by TLC). After completion, the reaction mixture was cooled to room temperature and acidified to pH 7 with acetic acid. The resulted yellow solid was collected by filtration, washed with ethanol (5–10 mL) and recrystallized from the mixture DMF:ethanol (1:2). Yield: 80%, mp 222–224 °C. IR (KBr), ν (cm^−1^): 3225 (NH), 3170 (C=C), 3104 (C=C), 1653 (C=O), 1643 (C=O), 1605 (C=C). ^1^H NMR (400 MHz, DMSO-*d_6_*): δ (ppm) 2.66 (s, 3H, CH_3_), 7.04 (t, *J* = 6.1 Hz, 1H, pyridine), 7.12 (d, *J* = 8.2 Hz, 1H, pyridine), 7.27–7.35 (m, 2H, arom.), 7.49 (t, *J* = 7.4 Hz, 1H, arom.), 7.51 (d, *J* = 15.4 Hz, 1H, =CH), 7.69 (d, *J* = 15.6 Hz, 1H, =CH), 7.78 (t, *J* = 7.7 Hz, 1H, arom.), 7.94 (t, *J* = 7.8 Hz, 1H, pyridine), 8.42 (d, *J* = 5.0 Hz, 1H, pyridine), 11.94 (s, 1H, NH). LCMS (ESI): *m*/*z* 309.9/312.0 (95.58%, [M+H]^+^). ^13^C NMR (100 MHz, DMSO-*d_6_*): δ (ppm) 18.7 (CH_3_), 111.6 (pyridine), 116.1 (^2^*J*_C-F_ = 25.2 Hz, arom.), 117.3 (pyridine), 122.2 (^3^*J*_C-F_ = 11.3 Hz, arom.), 123.8 (=CH), 125.1 (arom.), 127.4 (arom.), 129.7 (arom.), 132.3 (thiazole), 133.5 (=CH), 138.3 (pyridine), 146.6 (pyridine), 150.8 (pyridine), 159.8 (thiazole), 161.5 (thiazole) 161.6 (arom), 181.3 (C=O). LCMS (ESI): *m*/*z* 340/342 (100.00%, [M+H]^+^). Anal. Calc. for C_18_H_14_FN_3_OS: C 63.70%; H 4.16%; N 12.38%. Found: C 63.55%; H 4.20%; N 12.30%.

### 3.4. Synthesis of Ethyl 4-[[2-[1-(2-Fluorophenyl)-3-[4-methyl-2-(2-pyridylamino)thiazol-5-yl]-3-oxopropyl]sulfanylacetyl]amino]benzoate (***4***)

A mixture of compound **3** (10 mmol), 4-(2-mercaptoacetylamino)-benzoic acid ethyl ester (10 mmol) and *N*-methylpiperidine (20 mmol) were refluxed for 30 min in ethanol (15 mL). The process was monitored by TLC. After the synthesis was completed, the reaction mixture was cooled and the obtained solid products were filtered, washed with ethanol (5–10 mL) and recrystallized from the mixture DMF:ethanol (1:2). Yield: 81%, mp 178–180 °C. IR (KBr), ν (cm^−1^): 3264 (NH), 3166 (C=C), 3103 (C=C), 1718 (C=O), 1653 (C=O), 1633 (C=O),1604 (C=C). ^1^H NMR (400 MHz, DMSO-*d_6_*): 1.30 (t, *J* = 7.1 Hz, 3H, CH_3_), 2.52 (s, 3H, CH_3_), 3.29 (d, *J* = 14.5 Hz, 1H, CH_2_), 3.42 (d, *J* = 14.5 Hz, 1H, CH_2_), 3.59 (d, *J* = 7.3 Hz, 2H, CH_2_), 4.26 (q, *J* = 7.0 Hz, 2H, CH_2_), 4.89 (t, *J* = 7.2 Hz, 1H, CH), 7.02 (t, *J* = 6.1 Hz, 1H, pyridine), 7.09 (d, *J* = 8.3 Hz, 1H, pyridine), 7.11–7.19 (m, 2H, arom.), 7.22–7.29 (m, 1H, arom.), 7.53 (t, *J* = 7.7 Hz, 1H, arom.), 7.67 (d, *J* = 8.2 Hz, 2H, arom.), 7.76 (t, *J* = 7.8 Hz, 1H, pyridine.), 7.87 (d, *J* = 8.3 Hz, 2H, arom.), 8.37 (d, *J* = 5.1 Hz, 1H, pyridine), 10.38 (s, 1H, NH), 11.85 (s, 1H, NH). ^13^C NMR (100 MHz, DMSO-*d_6_*): δ (ppm) 14.2 (CH_3_), 18.5 (CH_3_), 35.7 (CH_2_), 37.7 (CH_2_), 46.3 (CH_2_), 60.4 (CH), 111.6 (pyridine), 115.5 (^2^*J*_C-F_ = 25.2 Hz, arom.), 117.2 (pyridine), 118.5 (arom.), 122.6 (arom.), 124.3 (arom.), 128.4 (^3^*J*_C-F_ = 17.4 Hz, arom.), 129.1 (arom.), 130.2 (arom.), 138.3 (pyridine), 143.2 (arom.), 146.5 (pyridine), 150.8 (pyridine), 159.9 (thiazole), 160.3 (thiazole), 161.1 (arom.), 165.3 (C=O), 168.1 (C=O), 188.7 (C=O). LCMS (ESI): *m*/*z* 577/579 (95.5%, [M+H]^+^). Anal. Calc. for C_29_H_27_FN_4_O_4_S_2_: C 60.19%; H 4.70%; N 9.68%. Found: C 60.25%; H 4.60%; N 9.80%.

### 3.5. Synthesis of {5-[5-(2-Fluorophenyl)-4,5-dihydro-1H-pyrazol-3-yl]-4-methylthiazol-2-yl}-(pyridin-2-yl)-amine (***5***)

To the mixture of compound **3** (10 mmol) in methanol (15 mL), hydrazine hydrate (10 mmol) was added slowly. The obtained mixture was refluxed for 1 h (monitored by TLC). The resulting yellow solid of **5** was filtered and recrystallized from the mixture DMF:ethanol (1:2). Yield: 76%, mp 188–190 °C. ^1^H NMR (400 MHz, DMSO-*d_6_*): 2.36 (s, 3H, CH_3_), 2.89 (dd, *J* = 16.3, 9.7 Hz, 1H, pyrazoline), 3.54 (dd, *J* = 15.9, 10.7 Hz, 1H, pyrazoline), 5.00 (td, *J* = 10.7, 2.8 Hz, 1H, pyrazoline), 6.91 (t, *J* = 6.2 Hz, 1H, pyridine), 7.01 (d, *J* = 8.3 Hz, 1H, pyridine), 7.14–7.23 (m, 1H arom.+ 1H (NH pyrazoline)), 7.24–7.36 (m, 2H, arom.), 7.51 (t, *J* = 6.8 Hz, 1H, arom.), 7.69 (t, *J* = 7.8 Hz, 1H, pyridine), 8.29 (d, *J* = 5.0 Hz, 1H, pyridine), 11.31 (s, 1H, NH). ^13^C NMR (100 MHz, DMSO-*d_6_*): δ (ppm) 17.5 (CH_3_), 42.2 (CH_2_, pyrazoline), 53.9 (CH, pyrazoline), 111.5 (pyridine), 115.8, (arom.), 116.6 (pyridine), 125.8 (arom.), 128.5 (arom.), 129.4, (arom.), 129.9 (arom.), 131.4 (thiazole), 138.2 (pyridine), 147.1 (pyridine), 150.6 (pyridine), 155.2 (pyrazoline), 159.7 (thiazole), 160.6 (thiazole) 161.9 (arom.). Anal. Calc. for C_18_H_16_FN_5_S: C 61.17%; H 4.56%; N 19.82%. Found: C 61.25%; H 4.65%; N 19.70%.

### 3.6. Synthesis of 1-{5-(2-Fluorophenyl)-3-[4-methyl-2-(2-pyridylamino)-thiazol-5-yl]-4,5-dihydropyrazol-1-yl}-ethanone (***6***)

To the mixture of compound **3** (10 mmol) in glacial acetic acid (10 mL), hydrazine hydrate (10 mmol) was added slowly. The obtained mixture was refluxed for 4 h (monitored by TLC). The resulting yellow solid of **6** was filtered and recrystallized from the mixture DMF:ethanol (1:2). Yield: 71%, mp 263–265 °C. IR (KBr), ν (cm^−1^): 3238 (NH), 3158 (C=C), 3090 (C=C), 1609 (C=O), 1597 (C=C). ^1^H NMR (400 MHz, DMSO-*d_6_*): 2.26 (s, 3H, CH_3_), 2.42 (s, 3H, CH_3_), 3.13 (dd, *J* = 17.3, 4.6 Hz, 1H, pyrazoline), 4.00 (dd, *J* = 17.3, 11.9 Hz, 1H, pyrazoline), 5.64 (dd, *J* = 11.8, 4.6 Hz, 1H, pyrazoline), 6.95–7.00 (m, 1H, pyridine), 7.05 (d, *J* = 8.3 Hz, 1H, pyridine), 7.11–7.23 (m, 3H, arom.), 7.29–7.35 (m, 1H, arom.), 7.73 (t, *J* = 6.9 Hz, 1H, pyridine), 8.35 (d, J = 4.0 Hz, 1H, pyridine), 11.58 (s, 1H, NH). ^13^C NMR (100 MHz, DMSO-*d_6_*): 17.7 (CH_3_), 22.1 (CH_3_), 43.5 (CH_2_, pyrazoline), 54.4 (CH, pyrazoline), 111.6 (pyridine), 116.2, (^2^*J*_C-F_ = 25.2 Hz, arom.), 117.1 (pyridine), 125.1 (arom.), 127.8 (arom.), 129.2, (^3^*J*_C-F_ = 13.8 Hz, arom.), 129.7 (^3^*J*_C-F_ = 7.6 Hz, arom.), 132.5 (thiazole), 138.6 (pyridine), 147.0 (pyridine), 150.3 (pyridine), 151.6 (pyrazoline), 159.5 (thiazole), 160.9 (thiazole) 161.5 (arom.), 167.3 (C=O). LCMS (ESI): *m*/*z* 394/396 (100.0%, [M+H]^+^). Anal. Calc. for C_20_H_18_FN_5_OS: C 60.75%; H 4.59%; N 17.71%. Found: C 60.90%; H 4.65%; N 17.60%.

### 3.7. Synthesis of (E)-3-(2-Fluorophenyl)-1-[4-methyl-2-(pyridin-1-ium-2-ylamino)thiazol-5-yl]-2-propen-1-one Methanesulfonate (***7***)

To the mixture of compound **3** (10 mmol) in dry tetrahydrofuran (15 mL), methanesulfonic acid (10 mmol) was added slowly. The white solid obtained was filtered, washed with tetrahydrofuran (5–10 mL), and dried at room temperature. Yield: 77%, mp 232–234 °C. IR (KBr), ν (cm^−1^): 3248 (NH), 3147 (C=C), 3059 (C=C), 1633 (C=O), 1619 (C=O), 1604 (C=C), 1375 (S=O). ^1^H NMR (400 MHz, DMSO-*d_6_*): 2.38 (s, 3H, CH_3_), 2.64 (s, 3H, CH_3_), 7.03 (t, *J* = 6.2 Hz, 1H, pyridine), 7.11 (d, *J* = 8.3 Hz, 1H, pyridine), 7.23–7.32 (m, 2H, arom.), 7.49 (t, *J* = 7.3 Hz, 1H, arom.), 7.50 (d, *J* = 15.3 Hz, 1H, =CH), 7.67 (d, *J* = 15.6 Hz, 1H, =CH) 7.78 (t, *J* = 7.8 Hz, 1H, arom.), 7.94 (t, *J* = 7.8 Hz, 1H, pyridine), 8.40 (d, *J* = 5.0 Hz, 1H, pyridine), 11.96 (brs, 1H, NH). ^13^C NMR (100 MHz, DMSO-*d_6_*): 18.7 (CH_3_), 45.8 (CH_3_), 111.7 (pyridine), 116.0, (arom.), 117.3 (pyridine), 122.2, (arom.), 123.8 (=CH), 125.1 (arom.), 127.6 (arom.), 129.7 (arom.), 132.5 (thiazole), 133.5 (=CH), 138.4 (pyridine), 146.6 (pyridine), 150.7 (pyridine), 158.9 (thiazole), 160.4 (thiazole) 161.5 (arom), 181.3 (C=O). LCMS (ESI): *m*/*z* 338/340 + 95.0 (100.0%, [M-H]^+^). Anal. Calc. for C_19_H_18_FN_3_O_4_S_2_: C 52.40%; H 4.17%; N 9.65%. Found: C 52.10%; H 4.30%; N 9.50%.

### 3.8. Crystal Structure Determination of (E)-3-(2-Fluorophenyl)-1-[4-methyl-2-(2-pyridylamino)-thiazol-5-yl]-2-propen-1-one Dimethylaminoformamide Solvate (3*DMF) and 1-{5-(2-fluorophenyl)-3-[4-methyl-2-(2-pyridylamino)-thiazol-5-yl]-4,5-dihydropyrazol-1-yl}-ethanone (***6***)

Compound **3** was recrystallized from the DMF by slow evaporation at room temperature. 

***Crystal data of compound *3**. C_18_H_14_FN_3_O_S_, C_3_H_7_NO, Mr = 412.48, triclinic, space group *P*$\bar{1}$, *a* = 7.3206(3), *b* = 11.2096(4), *c* = 12.4911(5) Å, *α* = 97.781(3), *β* = 100.510(4), *γ* = 102.036(3)^o^, *V* = 969.52(7) Å^3^, *Z* = 2 (*Z’* = 1), *D*_calc_ = 1.413 g/cm^3^, *µ* = 1.788 mm^−1^, T = 130.0(1) K. 

***Data collection for the compound *3.** A green block crystal (DMF) of 0.25 × 0.18 × 0.08 mm was used to record 7655 (Cu *K*α-radiation, *θ*_max_ = 76.66°) intensities on a Rigaku SuperNova Dual Atlas diffractometer [54] using mirror monochromatized Cu*K*α-radiation from a high-flux microfocus source (*λ* = 1.54184 Å). Accurate unit cell parameters were determined by the least-squares technique from the *θ* values of 6149 reflections, θ range 3.66–76.45°. The data were corrected for Lorentz, polarization and for absorption effects [54]. The 7655 total unique reflections (*R*_int_ = 0.0196) were used for structure determination.

Compound **6** was recrystallized from DMF-MeOH (1:2) mixture by slow evaporation at room temperature. 

***Crystal data of the compound *6.** C_20_H_18_FN_5_OS, Mr = 395.45, triclinic, space group *P*$\bar{1}$, *a* = 9.5524(3), *b* = 12.0251(4), *c* = 18.1215(5) Å, *α* = 87.574(2), *β* = 80.917(2), *γ* = 67.306(3)°, *V* = 1895.96(10) Å^3^, *Z* = 4 (*Z’* = 2), *D*_calc_ = 1.385 g/cm^3^, *µ* = 1.782 mm^−1^, T = 130.0(1) K.

***Data collection for the compound *6.** A colorless plate crystal (DMF-MeOH) of 0.26 × 0.21 × 0.03 mm was used to record 33876 (Cu *Kα*-radiation, *θ*_max_ = 76.59°) intensities on a Rigaku SuperNova Dual Atlas diffractometer [54] using mirror monochromatized CuKα-radiation from a high-flux microfocus source (*λ* = 1.54184 Å). Accurate unit cell parameters were determined by the least-squares technique from the *θ* values of 21,075 reflections, *θ* range 3.92–76.52°. The data were corrected for Lorentz, polarization and for absorption effects [54]. The 7861 total unique reflections (*R*_int_ = 0.0267) were used for structure determination. 

***Structure solution and refinement of the compounds *3 *and* 6.** The structure was solved by a dual space algorithm (SHELXT) [55] and refined against *F*^2^ for all data (SHELXL) [56]. The position of the H atom bonded to the N atom was obtained from the difference Fourier map and was refined freely. The remaining H atoms were positioned geometrically and were refined within the riding model approximation: C–H = 0.98 Å (CH_3_), 0.95 Å (*Csp^2^*H), and *U*_iso_(H) = 1.2*U_eq_*(C) or 1.5*U_eq_*(C) for methyl H atoms (compound **3**); C–H = 0.98 Å (CH_3_), 0.99 Å (CH_2_), 1.00 Å (*Csp^3^*H), 0.95 Å (*Csp^2^*H), and *U*_iso_(H) = 1.2*U_eq_*(C) or 1.5*U_eq_*(C) for methyl H atoms (compound **6**). The methyl groups were refined as rotatable rigid groups. Final refinement converged with: *R* = 0.0335 (for 4729 data with *F*^2^ > 4*σ*(*F*^2^), *w*R = 0.0907 (on *F*^2^ for all data), and *S* = 1.071 (on *F*^2^ for all data) (compound **3**); *R* = 0.0355 (for 7262 data with *F*^2^ > 4*σ*(*F*^2^), *w*R = 0.0978 (on *F*^2^ for all data), and *S* = 1.021 (on *F*^2^ for all data) (compound **6**). The largest difference peak and hole was 0.323 and −0.289 eÅ^3^ (compound **3**); 0.437 and −0.348 eÅ^3^ (compound **6**). 

The molecular illustration was drawn using ORTEP-3 for Windows [57]. Software used to prepare material for publication was WINGX [57], OLEX2 [58], and PLATON [59]. 

The supplementary crystallographic data are deposited at the Cambridge Crystallographic Data Centre (CCDC), 12 Union ROAD, Cambridge CB2 1EZ (UK) [phone, (+44) 1223/336-408; fax, (+44) 1223/336-033; e-mail, deposit@ccdc.cam.ac.uk; World Wide Web, http://www.ccdc.cam.ac.uk, accessed on 18 April 2021 (deposition no. CCDC 2193755, compound **3**, deposition no. CCDC 2193756, compound **6**)].

### 3.9. In Vitro Evaluation of the Anticancer Activity According to the DTP NCI Protocol

A primary anticancer assay was performed on a panel of approximately sixty human tumor cell lines derived from nine neoplastic diseases, in accordance with the protocol of the Drug Evaluation Branch, National Cancer Institute, Bethesda [31,32,33,34,35]. Tested compounds were added to the culture at a single concentration (10^−5^ M) and the cultures were incubated for 48 h. End point determinations were made with a protein binding dye, Sulphorhodamine B (SRB). The results for each tested compound were reported as the percentage of growth of the treated cells when compared to the untreated control cells. The percentage growth was evaluated spectrophotometrically versus controls not treated with test agents. The cytotoxic and/or growth inhibitory effects of the most active selected compounds were tested in vitro against the full panel of human tumor cell lines at concentrations ranging from 10^−4^ to 10^−8^ M. Then, 48 h continuous drug exposure protocol was followed and an SRB protein assay was used to estimate cell viability or growth. 

Using absorbance measurements [time zero (Tz), control growth in the absence of drug (C), and test growth in the presence of drug (Ti)], the percentage growth was calculated for each drug concentration. Percentage growth inhibition was calculated as:

[(Ti − Tz)/(C − Tz)] × 100 for concentrations for which Ti ≥ Tz,

[(Ti − Tz)/Tz] × 100 for concentrations for which Ti < Tz.

Dose response parameters (GI_50_, TGI, LC_50_) were calculated for each compound. Growth inhibition of 50% (GI_50_) was calculated from [(Ti − Tz)/(C − Tz)] × 100 = 50, which is the drug concentration resulting in a 50% lower net protein increase in the treated cells (measured by SRB staining) as compared to the net protein increase seen in the control cells. The drug concentration resulting in total growth inhibition (TGI) was calculated from Ti = Tz. The LC_50_ (concentration of drug resulting in a 50% reduction in the measured protein at the end of the drug treatment as compared to that at the beginning) indicating a net loss of cells following treatment was calculated from [(Ti − Tz)/ Tz] × 100 = −50. Values were calculated for each of these parameters if the level of activity was reached; however, if the effect was not reached or was excessive, the value for that parameter was expressed as more or less than the maximum or minimum concentration tested. The lowest values were obtained with the most sensitive cell lines. Compounds having GI_50_ values ≤ 100 μM were declared to be active.

### 3.10. Application of MTT Assay for Measuring Cells Viability

The antineoplastic action of the new heterocyclic derivatives was evaluated using MTT (3-[4,5-dimethylthiazol-2-yl]-2,5-diphenyl tetrazolium bromide) assay (Sigma-Aldrich, Burlington, MA, USA). The 4000 adherent or 15,000 suspension cells per well were seeded in 96-well plates in 100 µL DMEM (Sigma-Aldrich, Burlington, MA, USA) or RPMI-1640 (Sigma-Aldrich, Burlington, MA, USA), supplemented with 10% heat-inactivated fetal bovine serum (Sigma-Aldrich, Burlington, MA, USA), and incubated for 72 h at 37 °C in CO_2_-incubator with studied compounds at final concentrations of 1, 10, 50 µM. After incubation, 20 μL of MTT reagent (5 mg/mL) were added and incubated for the next 4 h. Crystals of formazan were dissolved in the dimethylsulfoxide, and the reaction absorbance was measured in accordance with the manufacturer’s recommendations by an Absorbance Reader BioTek ELx800 (BioTek Instruments, Inc., Winooski, VT, USA). The half maximal inhibitory concentration value (IC_50_) was calculated by GraphPad Prism 6 software (San Diego, CA, USA) using nonlinear regression [60].

### 3.11. Time-Dependent Viability Test in the MCF-7 Cell Line

Time-dependent viability data were obtained as a result of 24, 48, 72, and 124 h MTT tests performed on the MCF-7 cells treated with the compound **4** (10 μM).

### 3.12. Clonogenic Assay

MCF-7 cells were seeded in triplicate in 6-well plates (CytoONE, STARLAB International GmbH, Germany) at a density of 500 cells per well. After 24 h incubation, the compound **4** was added in 1 and 10 μM concentrations and cells were treated for 10 days. Colonies containing ≥ 50 cells were fixed with 50% (*v*/*v*) methanol in the PBS and stained with crystal violet (Sigma-Aldrich, Burlington, MA, USA). Stained colonies were counted and expressed as a fraction of the untreated control [61].

### 3.13. Soft Agar Drug Sensitivity Assay

Five hundred MCF-7 breast cancer cells in 0.5% sterile agar (Bacto-Agar, Difco Laboratories) were layered on a preformed 0.8% sterile agar layer using 24 well plate (CytoONE, STARLAB International GmbH, Hamburg, Germany). The cells in agar were treated with the compound **4** at concentrations of 1 and 5 µM/mL. Doxorubicin was used as a positive control. The 0.5% agar layer included DMEM medium containing 10% FBS, cells, and tested compounds. The 0.8% agar layer included DMEM medium. Colonies with a size of >50 µm were counted under the microscope and photographed after 12 days [62].

### 3.14. Application of Fluorescent Microscopy for Investigation of Morphological Changes in the KB3-1 Cells

KB3-1 cells were seeded in the 12-well plates (CytoONE, STARLAB International GmbH, Hamburg, Germany) on glass microscopic slides and incubated overnight. Studied compounds were added to cells in the following concentrations: the compounds **3**, **4** and cisplatin—5 µM/mL, doxorubicin—1 µM/mL. Cells were incubated for the next 48 h. Chromatin material of living and apoptotic cells was stained with the DNA-specific fluorescent dye Hoechst 33342 (Sigma-Aldrich, Burlington, MA, USA) and the DNA/RNA-specific fluorescent dye Propidium iodide (PI, Sigma, Burlington, MA, USA). Fluorochromes were added to cultured cells at final concentrations 0.2–0.5 μg/mL for Hoechst 33342 and 0.1 μg/mL—for PI. Images were made with a fluorescent Zeiss microscope (Carl Zeiss, Oberkochen, Germany). Magnification ×400. Microphotographs were additionally analyzed using ImagePro7N software [63].

### 3.15. Spectroscopic Assay for Measuring the Ability of Compounds to Act on DNA

Salmon sperm DNA (1.65 mg/mL, Sigma-Aldrich, Burlington, MA, USA) was diluted in a Milli-Q water at 4 °C for 24 h. The purity of the DNA solution was checked by measuring the absorbance ratio A260/A280 at NanoDrop N-1000 UV/VIS Spectrophotometer (Thermo Scientific, Wilmington, NC, USA). Tested compounds were dissolved in acetone (stock solution at 5 mM/mL). DNA denaturation step, followed by re-annealing in the presence of the compounds, was included. Following incubation of DNA and compound, KMnO_4_ was added to a final concentration of 0.3 mM, and the absorbance at 405 nm was measured (Absorbance Reader BioTek ELx800 (BioTek Instruments, Inc., Winooski, VT, USA) in different time periods up to 3 h. The concentrations 3, 6, 12, and 24 µM/mL were tested for each compound and the results were expressed as the change in absorbance (NetAbs, net A405) during the reaction with KMnO_4_ between time zero and selected time points (1, 2, 3 h) and calculated using the formula:

Net A405 nm = A405 nm of the Expt (time, min) − A405nm of the Expt (zero min)
− A405 nm of the Control (time, min) − A405 mm of the Control (zero min)

where Net A405 represented the level of oxidation of the DNA-chemical adduct. The Expt = Salmon sperm DNA + DNA binding agent + KMnO_4_. The Control = DNA binding agent + KMnO_4_.

Appropriate controls of DNA alone and compound alone were included and these Abs values were subtracted from the test sample to provide the net change in absorbance. DNA-binding compounds were generally defined as falling within the groups where the net change in absorbance between zero and various time points was >0.05 or <−0.05. DNA non-binding compounds were defined as such where the net change in absorbance between zero and various time points was in the range from 0.05 to −0.05 [41].

### 3.16. Annexin V Binding Assay

Apoptosis was determined, as described [42], using Annexin V-FITC binding by means of the FITC Annexin V Apoptosis Detection Kit II(BD Biosciences, San Diego, CA, USA) according to the manufacturer’s instruction. Cells (10,000 cell measured) were analyzed in a BD FACSCanto II flow cytometer (BD Biosciences, San Diego, CA, USA). Annexin V binds with high affinity to phosphatidylserine, and thus, can be used to identify cells in all stages of the programmed cell death. Propidium iodide (PI) exclusively stains cells with a disrupted cell membrane and can be used to identify late apoptotic and dead cells. Cells cultured in a drug-free medium were used as controls. Optimal parameter settings were found using a positive control (cells incubated with 3% formaldehyde in buffer during 30 min on ice). MCF-7 breast cancer cells were incubated for 24 h (37 °C, 5% CO_2_, 90–95% humidity) with the compounds **4** and **7** at a concentration of 5 µM. After incubation, in cells treated with the tested compounds as well as the controls, the culture medium was removed and cells were washed twice with cold PBS. Subsequently, the cells were resuspended in the Binding Buffer included in the detection Kit at a concentration of 1 × 10^6^ cells/mL. From each sample, 100 µL of cell suspension was taken and transferred to test tubes to which 5 µL of Annexin V-FITC and Propidium iodide (PI) were added. The contents of the test tubes were gently vortexed and incubated for 15 min at room temperature, protected from light. After the required time, the contents of the test tubes were made up to 500 µL with the Binding Buffer and immediately analyzed in a flow cytometer. The ratio of the apoptotic cells (early and late) was measured as: a percentage of cells from the upper right square (color red) to percentage of cells from the lower right square (color blue). Analysis was performed using the BD FACSCanto II flow cytometer, and the results were analyzed with FACSDiva software (both from BD Biosciences Systems, San Jose, CA, USA). The equipment calibration was performed using BD Cytometer Setup and Tracking Beads (BD Biosciences, San Diego, CA, USA).

### 3.17. Determination of Mitochondrial Membrane Potential (MMP)

Disruption of the MMP was assessed using the lipophilic cationic probe 5,5,6,6-tetrachloro-1,1,3,3-tetraethylbenzimidazolcarbocyanine iodide (JC-1 MitoScreen kit; BD Biosciences San Diego, CA, USA), as described previously [42]. The entire assay was performed according to the manufacturer’s instructions provided with the purchased kit. The MCF-7 cells were incubated for 24 h (37 °C, 5% CO_2_, 90–95% humidity) with the compounds **4** and **7** at a concentration of 5 µM. Briefly, unfixed MCF-7 cells were washed and resuspended in the PBS supplemented with the JC-1 dye. Then, cells were incubated for 15 min at room temperature (RT) in the dark, washed, and resuspended in the PBS for the immediate BD FACSCanto II flow cytometry analysis. The percentage of cells with disrupted MMP was calculated in the FACSDiva software (both from BD Biosciences Systems, San Jose, CA, USA). The equipment calibration was performed using BD Cytometer Setup and Tracking Beads (BD Biosciences, San Diego, CA, USA).

### 3.18. PARP-1 Inhibition In Vitro

To determine PARP inactivation, the MCF-7 cells were pretreated for 2 h in the non-toxic concentrations of 5, 10, 15 µM of the Fluzoparib (Med Chem Express, Monmouth Junction, NJ, USA), a potent PARP1 inhibitor used in the chemotherapy [64]. Then, the medium was removed and increasing doses of the compounds **3** and **4** were added and cells were incubated for the next 72 h. The IC_50_ values of compounds were determined by the MTT assay, as described [60].

### 3.19. Study of Chemical Synthetic Lethality for Breast cAncer: New Synthesized PARP1 Inhibitor ***4*** Combined with BRCA1 and MGMT Inhibitors

Using the BRCA1 and MGMT inhibitors, we simulated the deficiency of these enzymes in the MCF-7 cells [49]. Breast cancer cells were preincubated with the MGMT and BRCA1 inhibitors used 1, 5, 10, and 15 µM for 3 h with Lomeguatrib (MGMT inhibitor, Med Chem Express, Monmouth Junction, NJ, USA) [65] and for 30 min—with Bractoppin (BRCA1 inhibitor, Med Chem Express, Monmouth Junction, NJ, USA) [66]. Then, the medium was removed and compound **4** was added at 1, 5, and 10 µM. The cell viability was measured after 72 h of incubation using the MTT assay, as mentioned above.

### 3.20. Statistical Data Analysis

The results were analyzed and illustrated with GraphPad Prism 6 (GraphPad Software, San Diego, CA, USA), and presented as a mean (M) ± standard deviation (SD) of 3 parallels. Statistical evaluation was performed using a two-way ANOVA analysis followed by Tukey’s multiple comparisons test. Data were considered statistically significant if ** *p* < 0.01, *** *p* < 0.001, **** *p* < 0.0001 [63].

### 3.21. Molecular Docking Studies

Docking models were based on 3D crystal structures of the PARP1 [67] and PARP2 [68] were retrieved from Protein Data Bank (PDB). Hyperchem software was used for the 3D structure generation using Molecular Mechanics MM+ and Semi-Empirical Quantum Technique for energy minimization procedure. The docking protocol was confirmed by the removing of the co-crystallized inhibitor from the protein and docking it on the same binding pockets. Protein’s structures were modified by adding of the polar hydrogens and Kollman charges during protein preparation by Autodock Tools V.4.2.6 graphical user interface [69]. The cuboid grid box with the size 60 × 60 × 60 was used for the computation with the purpose to embrace all the minimized inhibitors in all three dimensions. The Lamarckian genetic algorithm (LGA) [70] was used to generate conformations of ligands within the binding site, with a number of the GA runs 50, the initial population size of 300 individuals, with a maximum number of 250,000 energy evaluations, 150,000 generations with a mutation rate of 0.02, and a crossover rate of 2 points. The rigidity parameters were set for the receptor, keeping the ligand flexible. The lowest binding energy conformations were selected for a comparison with the docked results of the reference ligands. For estimating of possible inhibition activities of the proposed ligands, we also compared binding energies and estimated inhibition constant Ki, either of native ligands from the downloaded 3D structures or the Fluzoparib and Talazoparib structures in the cross-docking studies. Root mean square deviation (RMSD) was calculated during the validation procedure RMSD value of ≤2 Å was considered relevant for the prediction of the binding orientation of interaction energies of the ligands [71]. Discovery Studio Visualizer v.21.1. was used for the visualization and interpretation of the received data.

## 4. Conclusions

Novel pyridine-thiazole hybrid molecules were synthesized and tested on a panel of tumor and pseudo-normal cell lines. Based on specified biological and chemical advantages, the most active compound **4** was selected. The presented data highlight the potential of this compound as PAPR1/2 inhibitor, apoptosis inducer, and inhibitor of cell growth in a colony formation assay. This pyridine-thiazole hybrid molecule interacts with PAPRs in a different from other PARP inhibitors way. This compound may be a promising chemical probe to investigate anticancer potential, alone and in a combination with DNA repair protein inhibitors or DNA damaging agents.

## Figures and Tables

**Table 1:** Hydrogen-bond geometry (Å, °) for (3*DMF).

*D*—H···*A*	*D*—H	H···*A*	*D*···*A*	*D*—H···*A*
N6―H6···O24	0.878(18)	1.966(18)	2.8418(15)	175.1(15)
C16―H16···F1B	0.95	2.12	2.806(7)	128
C25―H25···N3	0.95	2.56	3.2011(18)	125

**Table 2:** Hydrogen bonds and C―H···*Cg* interactions (Å, °) in the crystal structure of **6**.

*D*—H···*A*	*D*—H	H···*A*	*D*···*A*	*∠ D—*H*···A*
N6A―H6A···O20B	0.88(2)	1.90(2)	2.7621(16)	168(2)
N6B―H6B···O20A ^i^	0.85(2)	1.93(2)	2.7784(16)	176(2)
C21B―H21B···N3A	0.98	2.53	3.490(2)	165
C21A―H21E···N3B ^ii^	0.98	2.55	3.489(2)	161
C—H···*Cg*	C—H	H···*Cg*	C···*Cg*	∠ C—H···*Cg*
C9A―H9A···*Cg*1 ^iii^	0.95	2.86	3.6228(16)	138
C18A―H18C···*Cg*2 ^iv^	0.99	2.70	3.4533(15)	133

Symmetry codes: (i) *x*, −1 + *y*, 1 + *z*, (ii) *x*, 1 + *y*, −1 + *z*, (iii) −1 + *x*, *y*, *z*, (iv) 1 − *x*, 1 − *y*, −*z*; *Cg*1 = thiazole ring, *Cg*2 = phenyl ring.

**Table 3:** Anticancer Screening Data in Concentration 10 μM.

Comp.	Mean Growth, %	Range of Growth, %	Most Sensitive Cell Lines/Growth %	Positive Cytostatic Effect ^1^	Positive Cytotoxic Effect ^2^
**4**	−2.78	−76.78–41.12	NCI-H460 (Non-Small Cell Lung Cancer)/−40.69 NCI-H522 (Non-Small Cell Lung Cancer)/−22.73 Colo 205 (Colon Cancer)/−34.08 HCT-15 (Colon Cancer)/−42.04 HT29 (Colon Cancer)/−28.32 KM12 (Colon Cancer)/−59.82 SF-539 (CNS Cancer)/−27.26 U251 (CNS Cancer)/−70.41 LOX IMVI (Melanoma)/−76.78 SK Mel-2 (Melanoma)/−31.22	35/60	25/60
**5**	50.64	−18.90–118.83	CCRF-CEM (Leukemia)/19.12 HL-60(TB) (Leukemia)/4.96 K-562 (Leukemia)/15.13 SR (Leukemia)/12.44 NCI-H522 (Non-Small Cell Lung Cancer)/−18.90 MDA-MB-435 (Melanoma)/−17.91 MDA-MB-468 (Brest Cancer)/−13.74	25/60	3/60
**6**	51.48	12.53–95.32	K-562 (Leukemia)/15.62 SR (Leukemia)/12.53 SK Mel-5 (Melanoma)/17.75 T-47D (Brest Cancer)/14.27	32/59	0/59

^1^ Ratio between a number of cell lines with percent growth from 0 to 50 and a total number of cell lines. ^2^ Ratio between a number of cell lines with percent growth of <0 and a total number of cell lines.

**Table 4:** Influence of Compound **4** on the Growth of Individual Tumor Cell Lines.

Disease	Cell Line	GI_50_, μM	SI
Leukemia	CCRF-CEM	18.6	1.70
	HL-60(TB)	21.4	1.48
	K-562	30.4	1.04
	MOLT-4	32.2	0.98
	RPMI-8226	8.29	3.82
	SR	24.1	1.32
	**MG_MID**	**22.5**	**1.41**
Non-Small Cell Lung Cancer	A549/ATCC	75.7	0.42
	EKVX	19.0	1.67
	HOP-62	37.5	0.85
	HOP-92	12.5	2.54
	NCI-H226	2.52	12.6
	NCI-H23	22.7	1.40
	NCI-H322M	25.4	1.25
	NCI-H460	31.2	1.02
	NCI-H522	21.6	1.45
	**MG_MID**	**27.6**	**1.14**
Colon Cancer	COLO 205	>100	0.32
	HCC-2998	23.1	1.37
	HCT-116	23.0	1.38
	HCT-15	25.8	1.23
	HT29	58.3	0.54
	KM12	55.6	0.57
	SW-629	75.3	0.42
	**MG_MID**	**51.6**	**0.61**
CNS Cancer	SF-268	43.1	0.74
	SF-295	32.2	0.98
	SF-539	32.5	0.98
	SNB-19	36.9	0.86
	SNB-75	3.58	8.85
	U251	47.8	0.66
	**MG_MID**	**32.7**	**0.97**
Melanoma	LOX IMVI	0.312	101.6
	MALME-3M	25.4	1.25
	M14	19.4	1.63
	MDA-MB-435	26.3	1.21
	SK-MEL-2	44.9	0.71
	SK-MEL-28	38.7	0.82
	SK_MEL-5	18.6	1.70
	UACC-257	38.4	0.83
	UACC-62	16.1	1.97
	**MG_MID**	**25.3**	**1.25**
Ovarian Cancer	IGROV-1	12.1	2.62
	OVCAR-3	31.7	1.00
	OVCAR-4	66.1	0.48
	OVCAR-5	74.4	0.43
	OVCAR-8	53.6	0.59
	NCI/ADR-RES	24.1	1.32
	SK-OV-3	26.0	1.22
	**MG_MID**	**41.1**	**0.77**
Renal Cancer	786-0	24.8	1.28
	A498	69.2	0.33
	ACHN	45.6	0.70
	CAKI-1	11.5	2.76
	RXF 393	2.59	12.2
	SN12C	28.0	1.13
	TK-10	82.3	0.39
	UO-31	3.19	9.94
	**MG_MID**	**33.4**	**0.95**
Prostate Cancer	PC-3	23.8	1.33
	DU-145	67.2	0.47
	**MG_MID**	**45.5**	**0.70**
Breast Cancer	MCF-7	15.4	2.06
	MDA-MB-231/ATCC	19.3	1.64
	HS 578T	14.9	2.13
	BT-549	14.7	2.16
	T-47D	18.0	1.76
	MDA-MB-468	4.24	7.48
	**MG_MID**	**14.4**	**2.20**
**MG_MID**		**31.7**	

**Table 5:** The IC_50_ values of screened compounds towards a panel of tumor and pseudo-normal cell lines. N/D—not determined (72 h of incubation, µM, M ± SD).

Comp./Cell Line	1	2	3	4	5	6	7	Doxorubicin
HCT-116	>50	>50	2.90 ± 1.03	6.11 ± 0.24	>50	>50	7.27 ± 0.27	0.90 ± 0.09
HCT-116 p53 (−/−)	>50	>50	2.81± 0.16	3.43 ± 0.90	34.54 ± 1.02	29.12 ± 1.45	N/D	1.19 ± 1.30
MCF-7	>50	>50	1.86 ± 0.90	2.79 ± 1.31	>50	45.37 ± 2.20	4.30 ± 0.98	1.04 ± 0.80
Jurkat	>50	>50	5.20 ± 0.10	5.36 ± 0.16	>50	>100	N/D	1.17 ± 0.12
HL-60	N/D	N/D	0.57 ± 0.35	N/D	N/D	N/D	>10	0.38 ± 0.50
A549	>50	>50	4.56 ± 0.22	8.05 ± 0.24	33.13 ± 1.32	63.35 ± 3.81	N/D	2.00 ± 0.35
SK-OV-3	>50	>50	>50	7.87 ± 0.23	>50	39.59 ± 2.37	N/D	3.20 ± 0.10
KB3-1	>50	38.01 ± 1.90	7.42 ± 0.22	7.63 ± 0.38	13.08 ± 0.52	24.88 ± 0.74	3.96 ± 1.23	4.00 ± 1.30
J774.2	>50	>50	>50	>50	>50	>50	N/D	1.74 ± 1.10
HEK-293	N/D	N/D	>50	>50	45.36 ± 2.26	N/D	N/D	1.29 ± 0.87
HaCaT	>50	>50	>50	>50	>50	>50	N/D	>10
BALB-3T3	N/D	N/D	7.18 ± 0.21	7.79 ± 0.38	34.16 ± 1.70	N/D	N/D	3.18 ± 0.25

**Table 6:** Docking scores of the studied compounds.

Compound	PARP1 (PDB 7KK4)		PARP2 (PDB 4PJV)	
	Binding Energy	Inhibition Constant Ki, nM	Binding Energy	Inhibition Constant Ki, nM
**3**	−7.84	1790	−8.08	1190
**4**	−9.95	50.82	−9.19	33.94
**5**	−8.75	384.91	−8.93	283.37
**6**	−9.32	147.71	−8.70	421.20
Fluzoparib	−11.88	1.97	−10.90	10.25
Olaparib	−12.72	0.473	−11.39	4.46
Talazoparib	−9.51	107.62	−9.98	48.61

## Data Availability

Not applicable.

## Supplementary Materials

The following supporting information can be downloaded at: https://www.mdpi.com/article/10.3390/molecules27196219/s1, Figures S1–S14: copies of ^1^H, ^13^C NMR and LC-MS spectra; Figures S15–S18: NCI protocols of anticancer activity for compound **4**, **5**, and **6**.

## Sample Availability

Samples of compounds **1**–**7** are available from the authors.
