# Supplementary material for: Development of Novel Pyridine-Thiazole Hybrid Molecules as Potential Anticancer Agents
Source: Molecules. 2022 Sep 21;27(19):6219. doi: 10.3390/molecules27196219 (PMC9570594; doi:10.3390/molecules27196219)
Supplement: Supplementary file 1 [file molecules-27-06219-s001.zip › molecules-1906381-supplementary.pdf]

Supplementary information

Table of Contents

Copies of ^{1}H, ^{13}C NMR, LC-MS and IR spectra of pyridine-thiazole derivatives .. S2

NCI protocols for compounds **4**, **5**, and **6** ………………………………………... S19

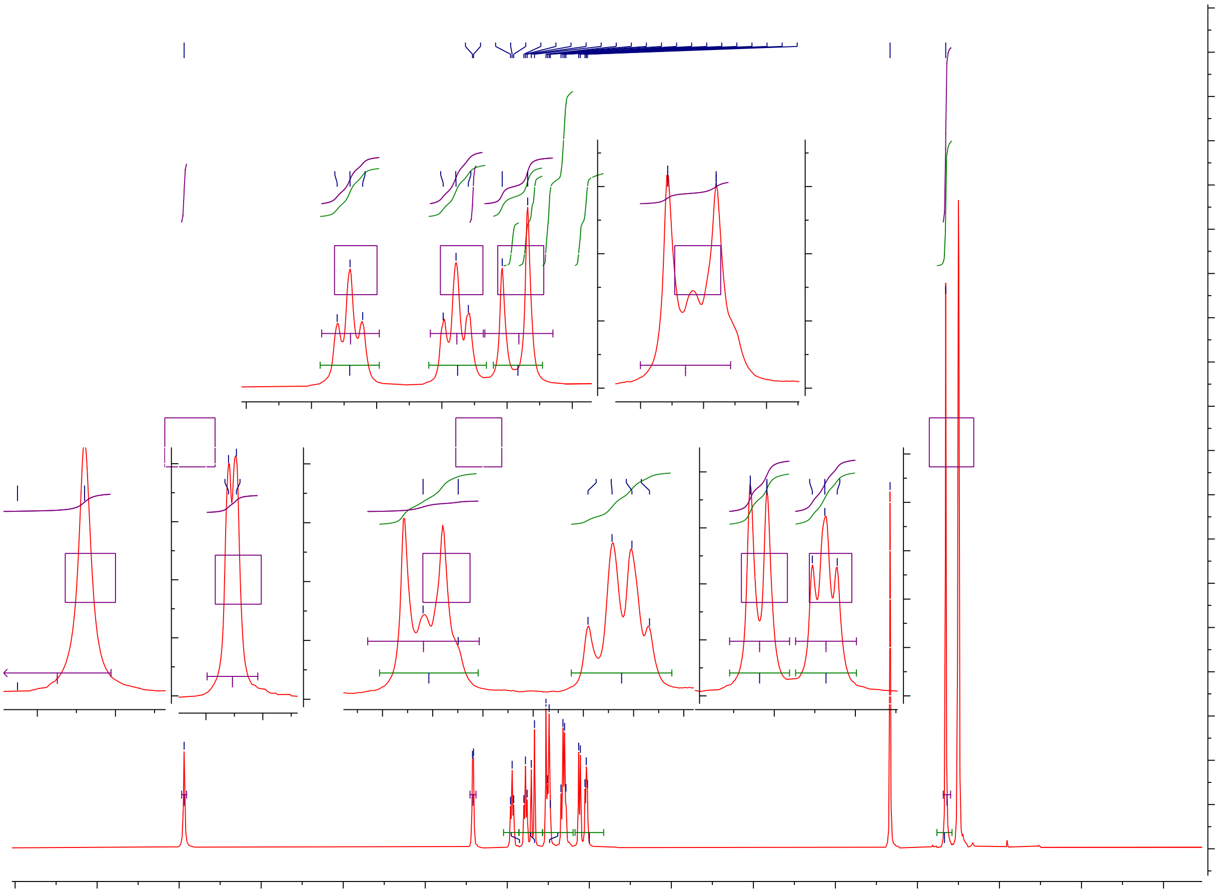

Figure S1. ^{1}H NMR Spectrum of compound **3**.

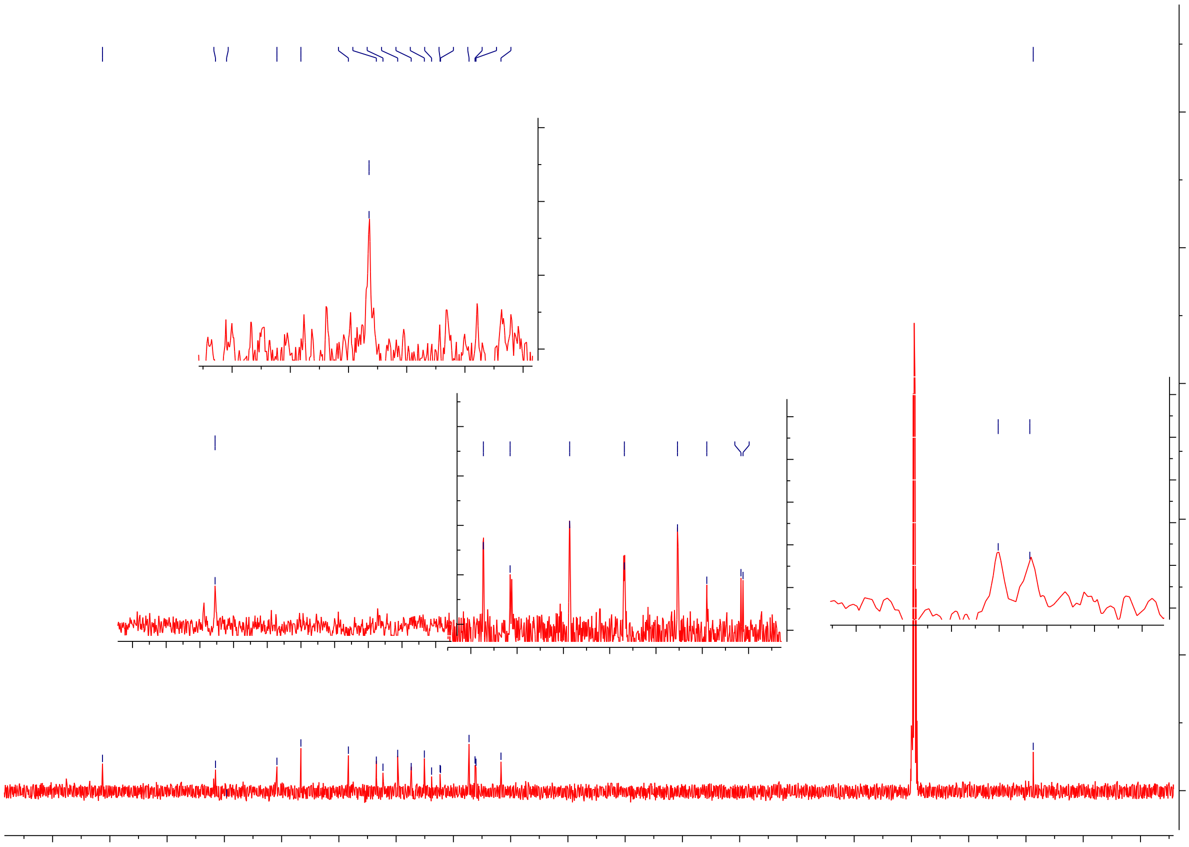

Figure S2. ^{13}C NMR Spectrum of compound **3**.

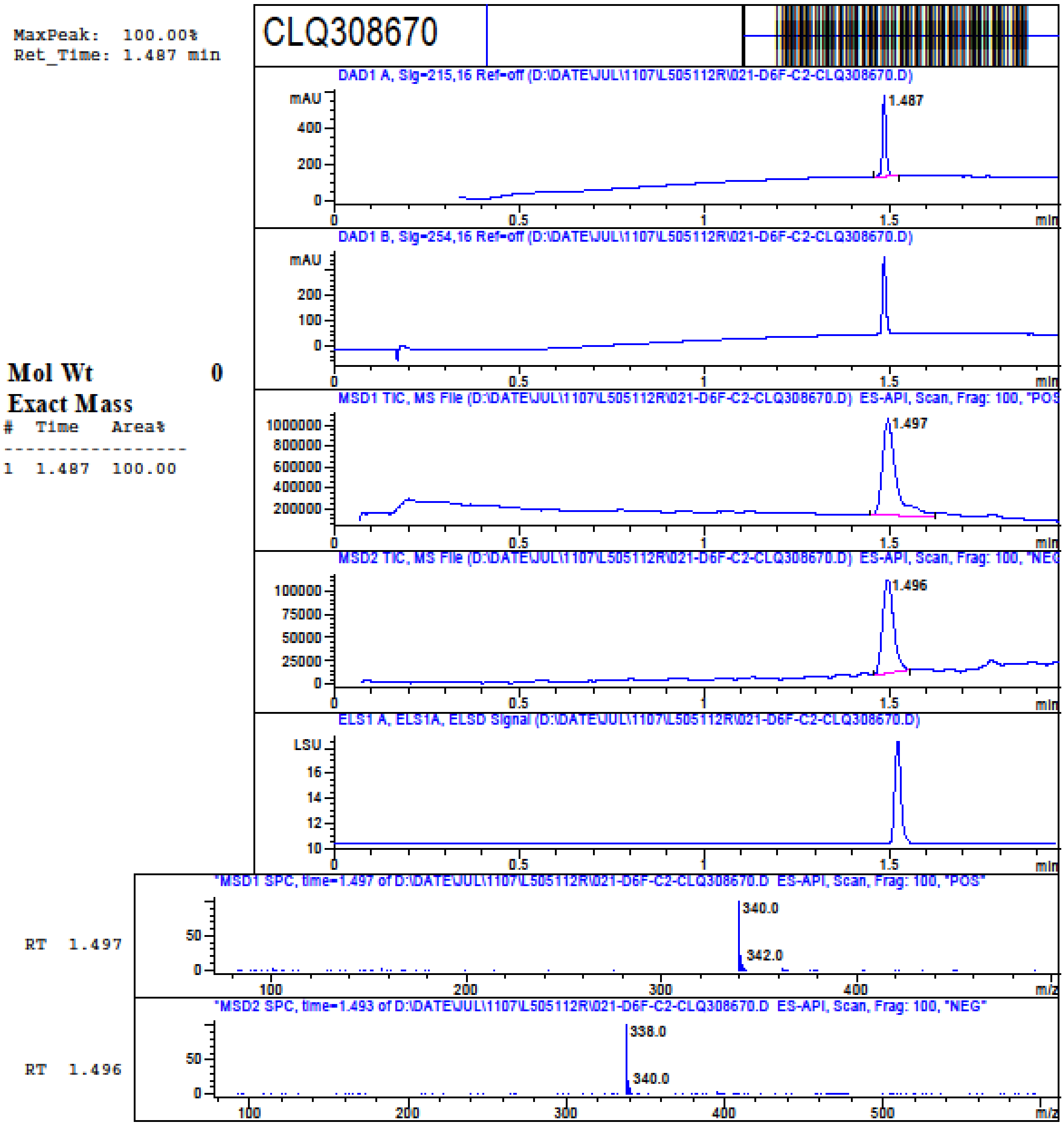

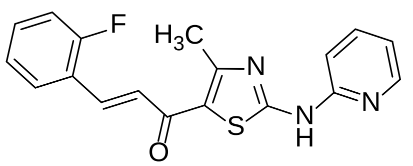

3

Molecular Weight: 339,39

Figure S3. LCMS Spectrum of compound **3**.

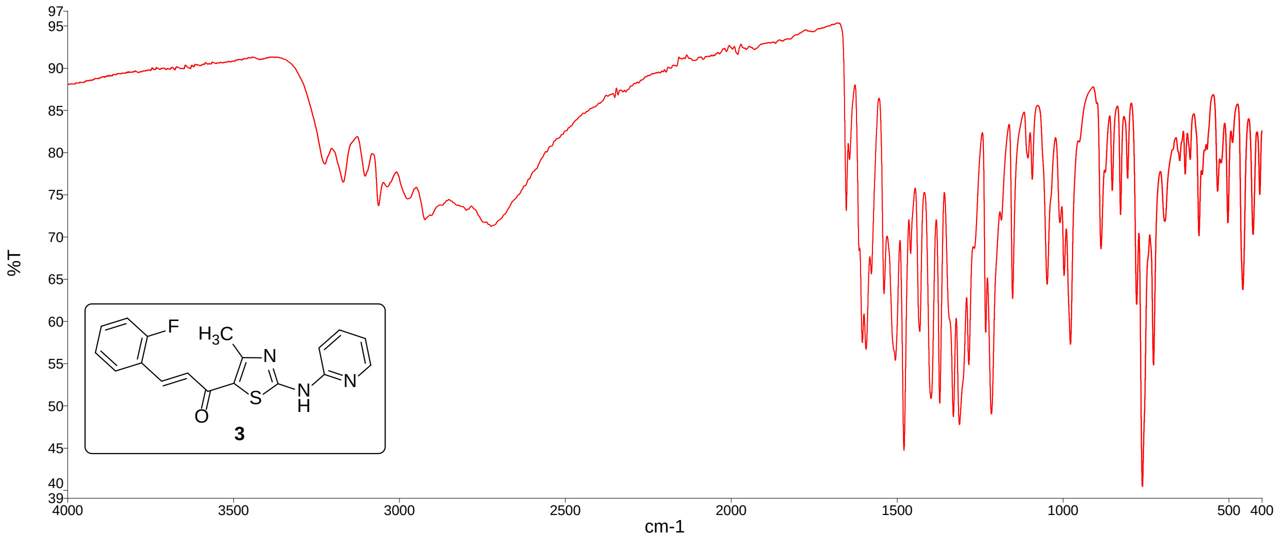

Figure S4. IR Spectrum of compound **3**.

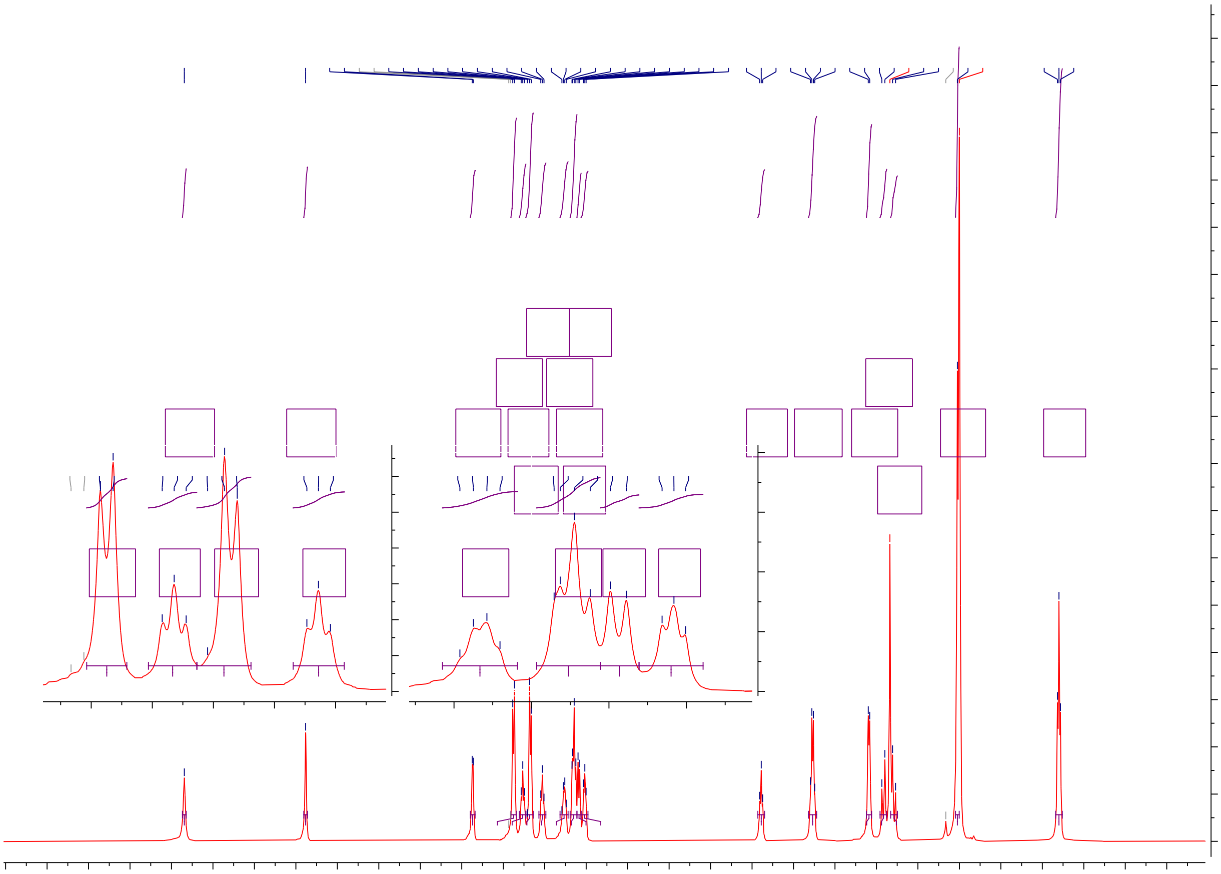

Figure S5. ^{1}H NMR Spectrum of compound **4**.

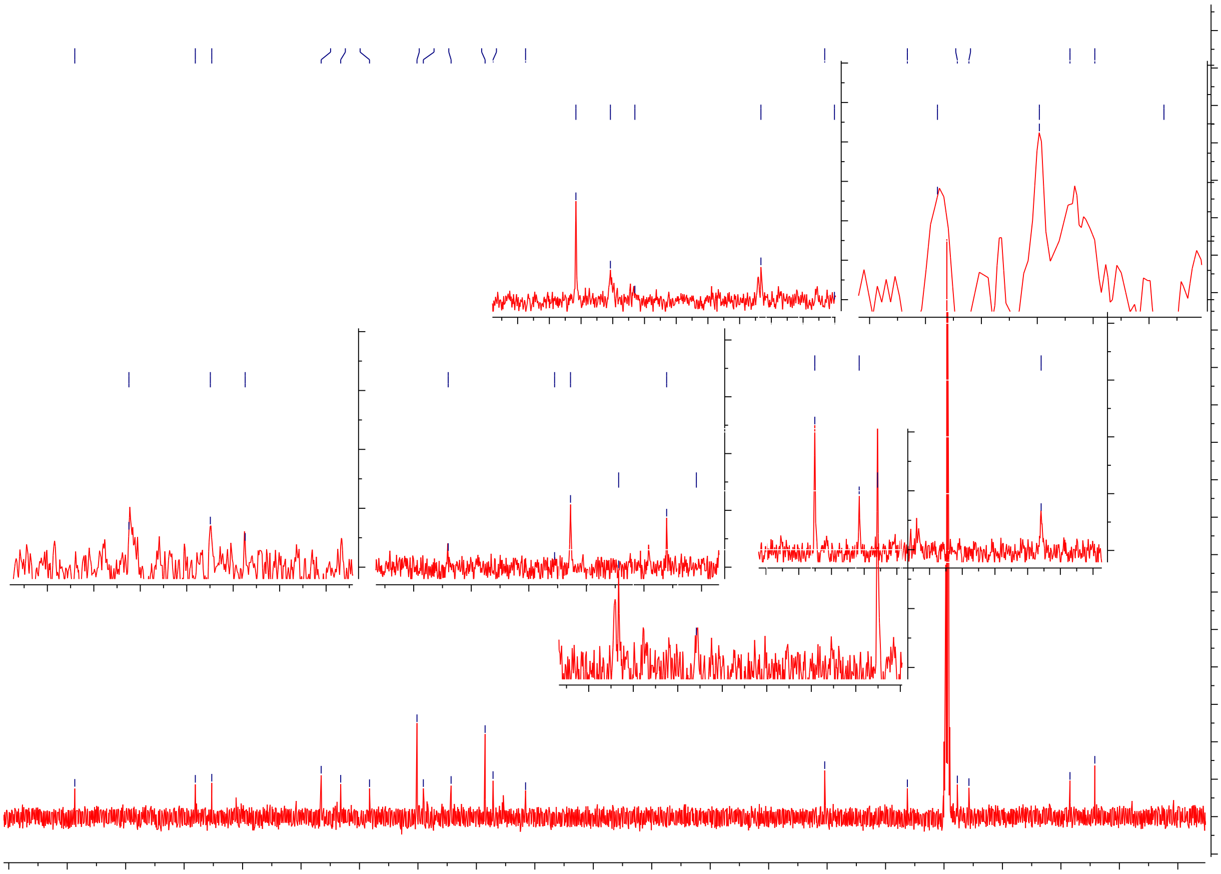

Figure S6. ^{13}C NMR Spectrum of compound **4**.

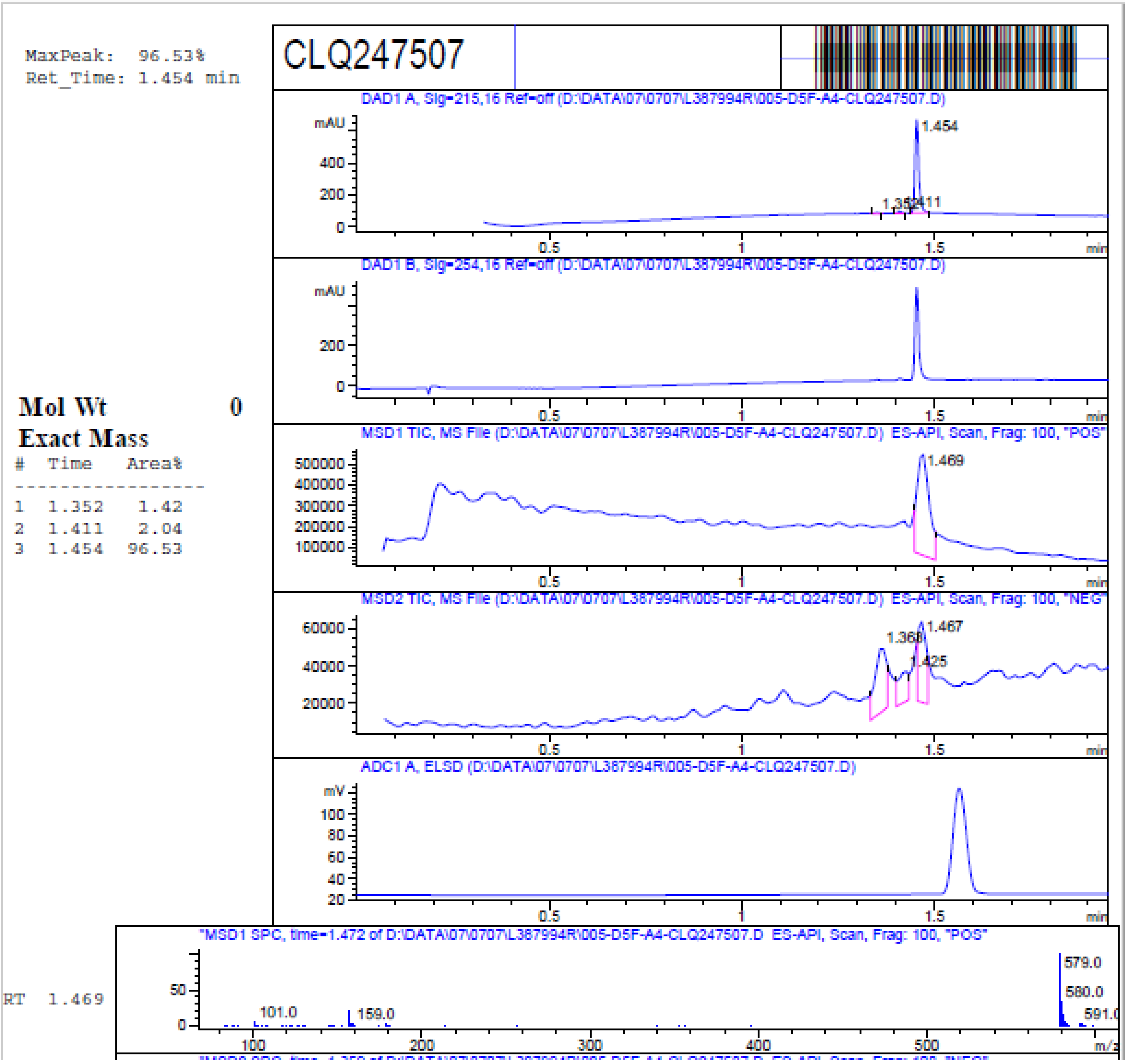

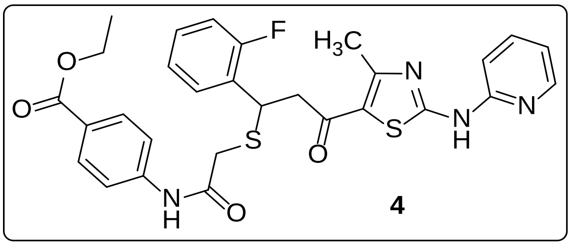

Molecular Weight: 578,68

Figure S7. LCMS Spectrum of compound **4**.

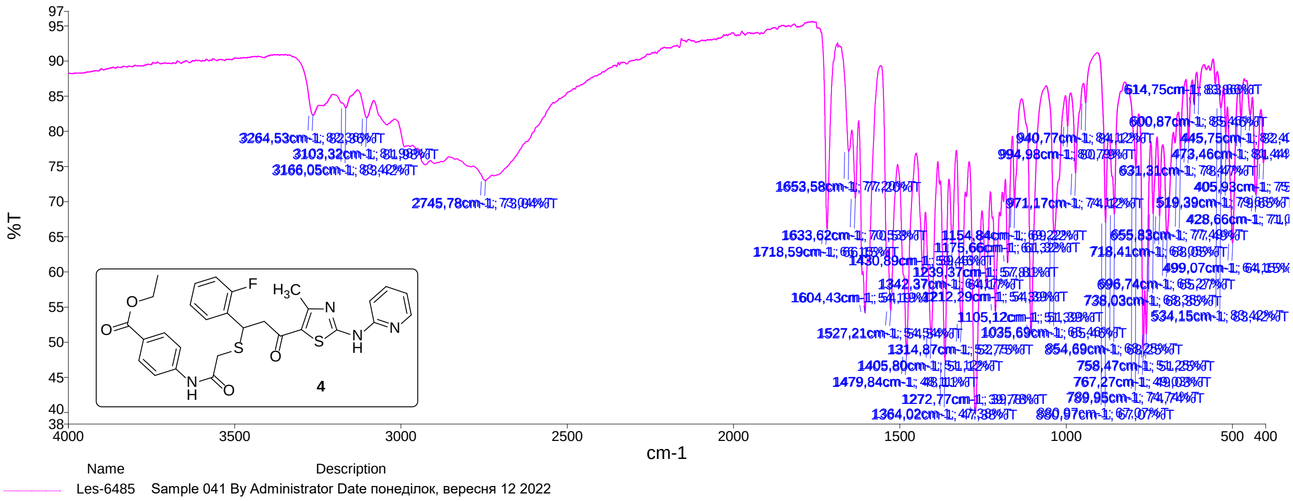

Figure S8. IR Spectrum of compound **4**.

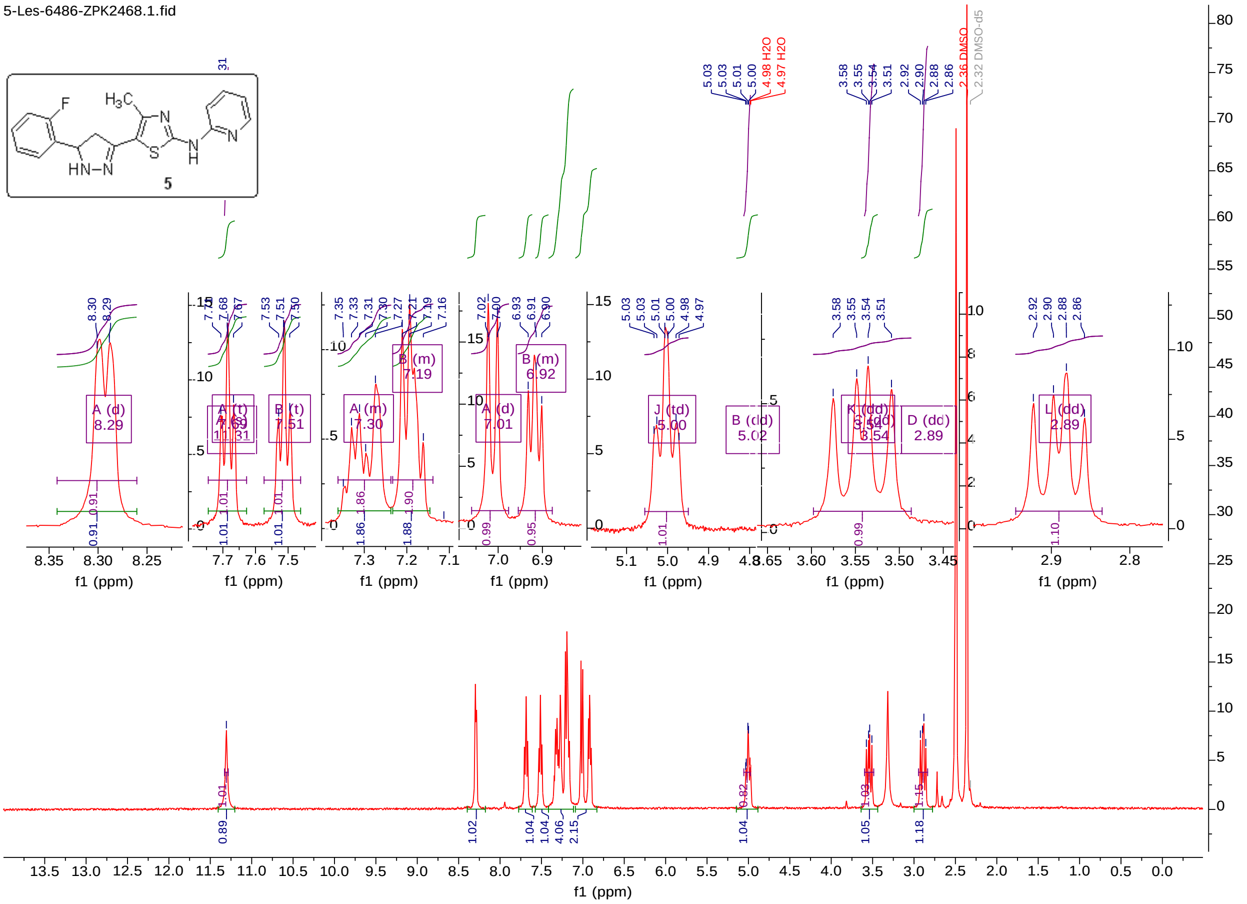

Figure S9. ^{1}H NMR Spectrum of compound **5**.

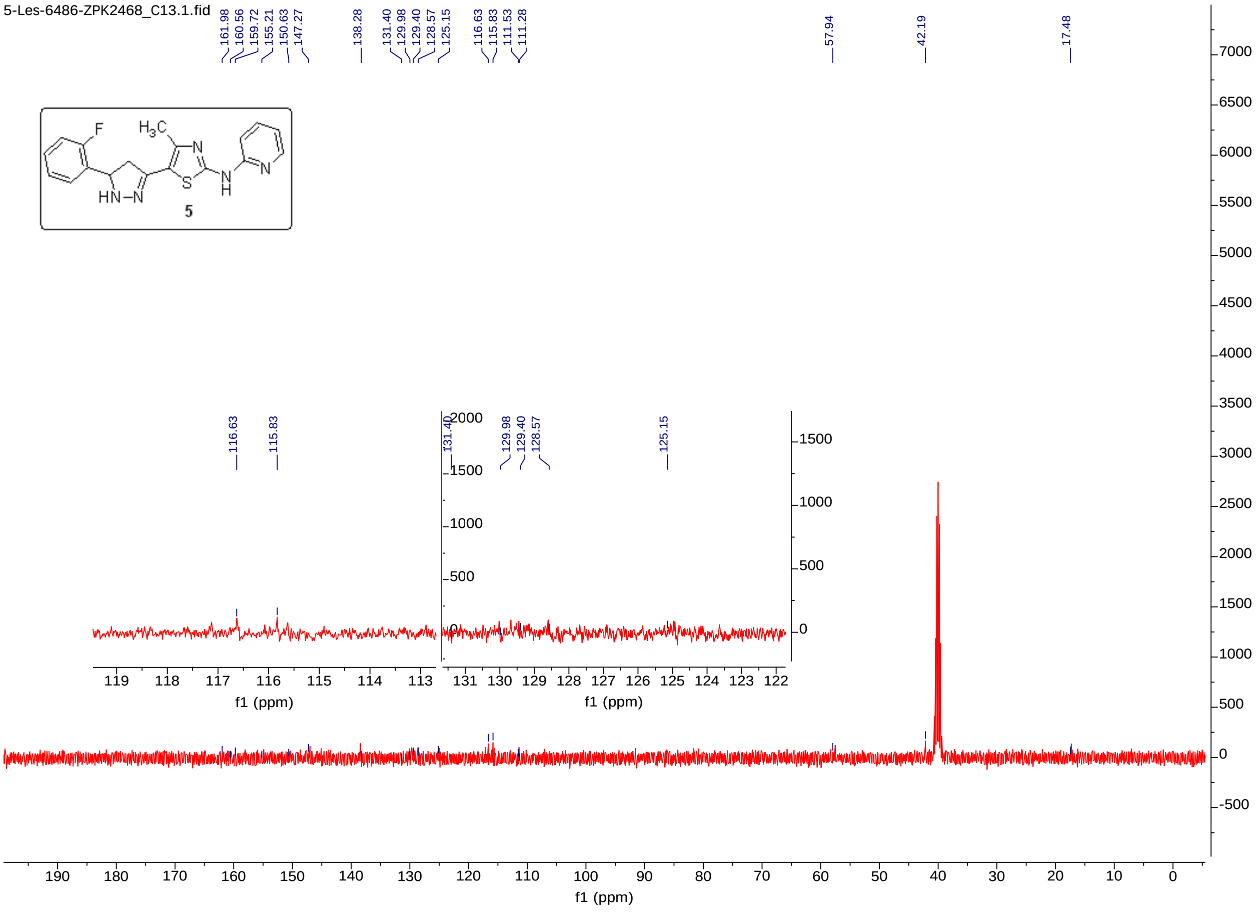

Figure S10. ^{13}C NMR Spectrum of compound **5**.

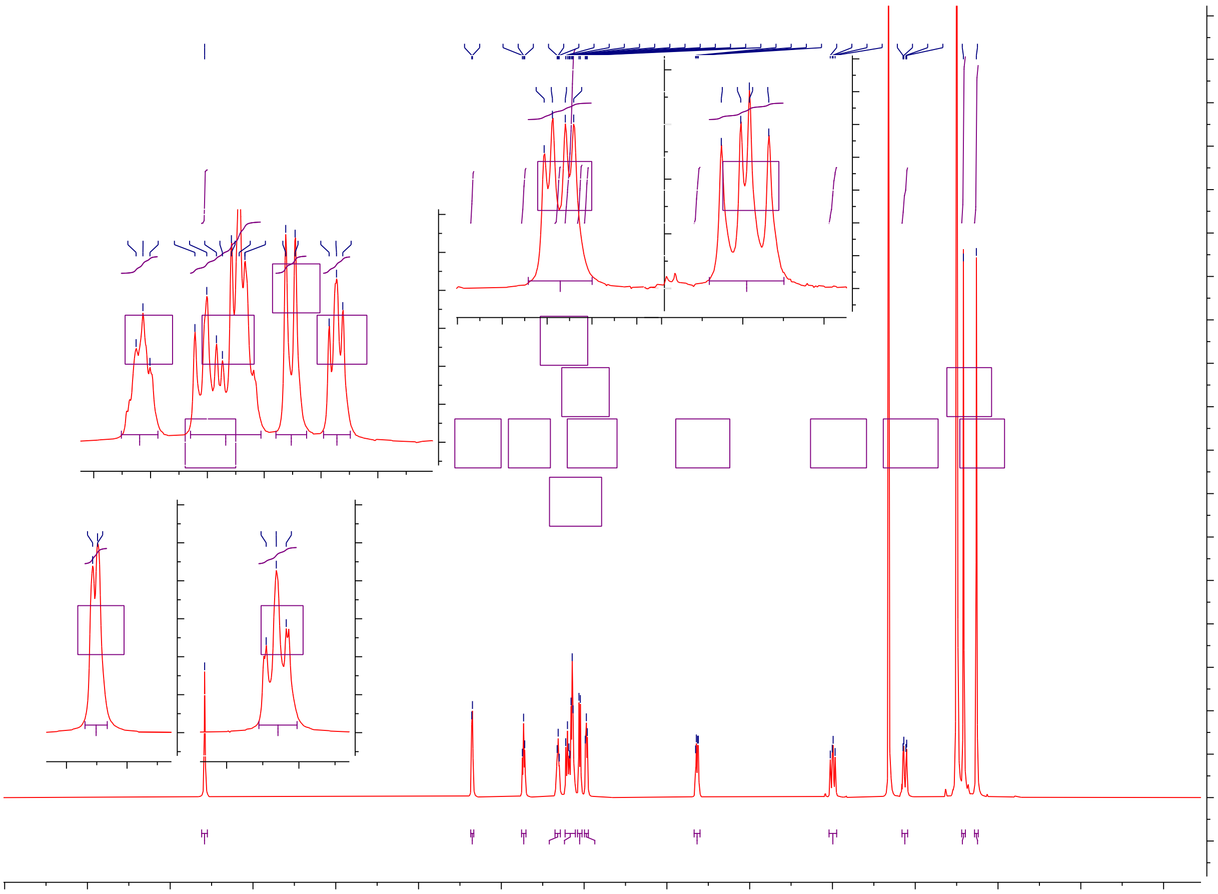

Figure S11. ^{1}H NMR Spectrum of compound **6**.

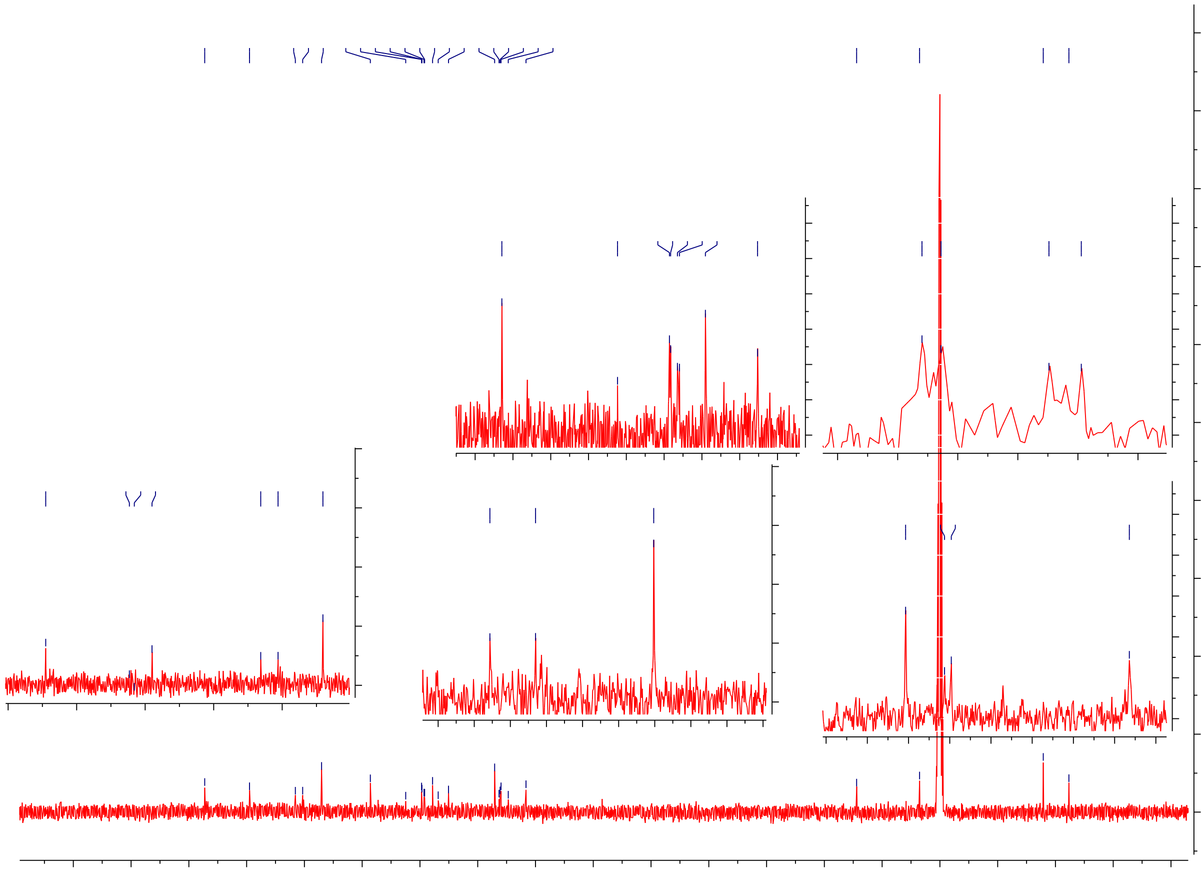

Figure S12. ^{13}C NMR Spectrum of compound **6**.

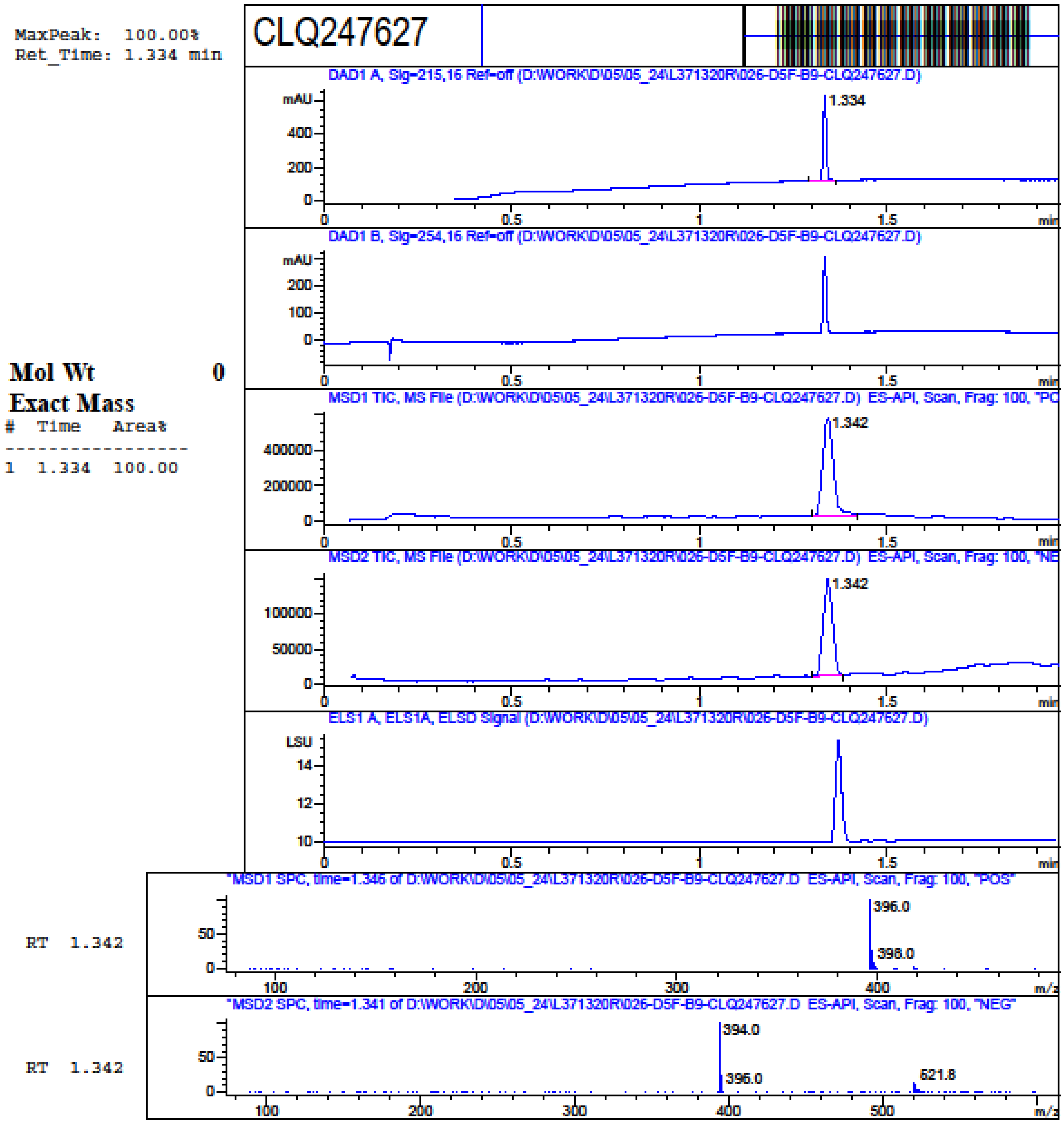

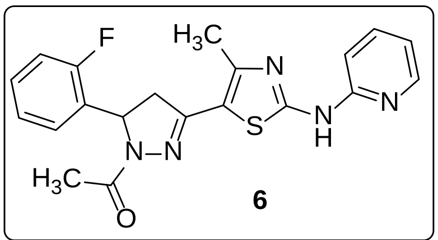

6

Molecular Weight: 395,46

Figure S13. LCMS Spectrum of compound **6**.

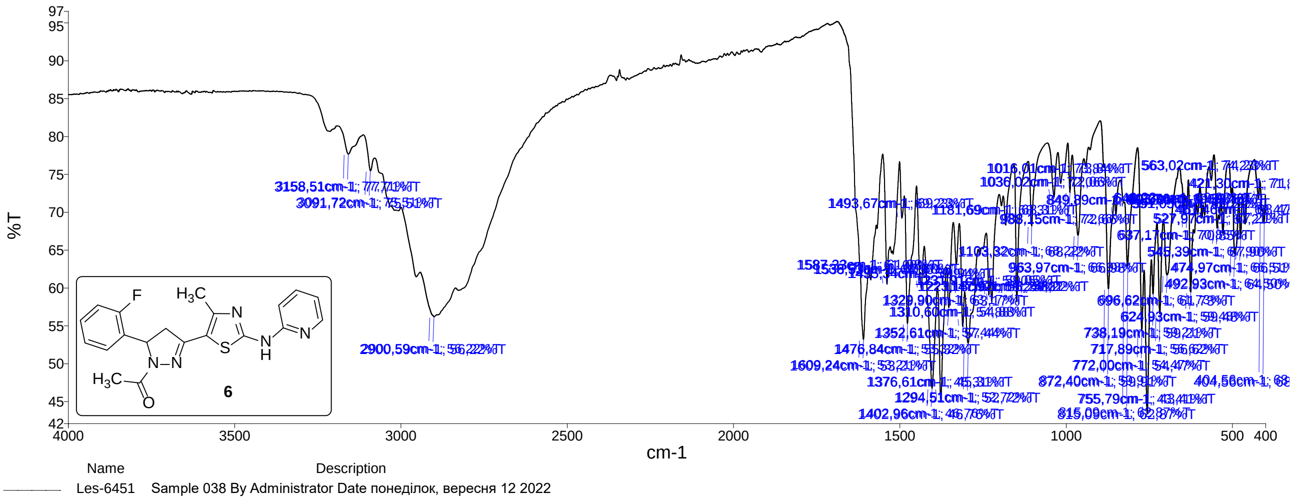

Figure S14. IR Spectrum of compound **6**.

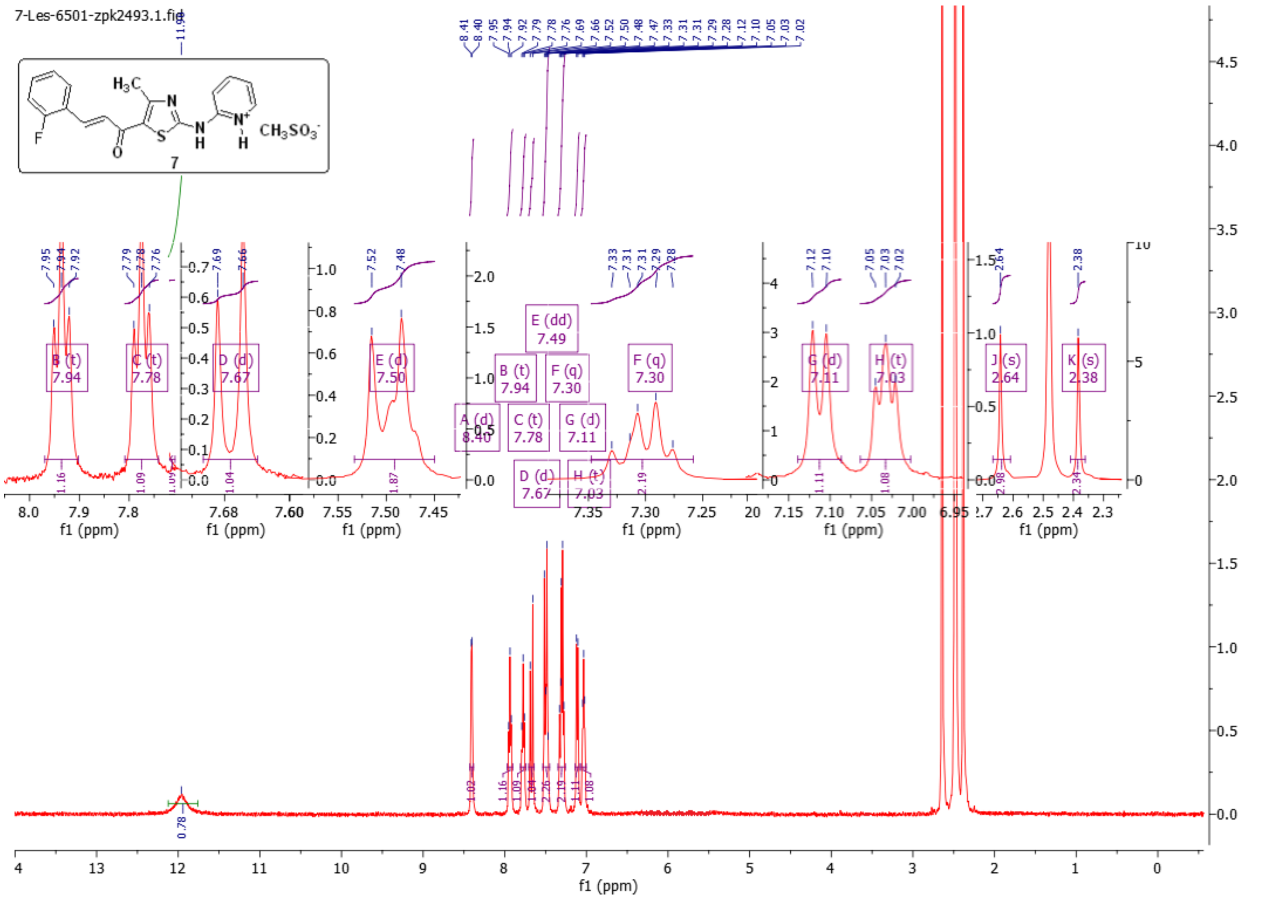

Figure S15. ^{1}H NMR Spectrum of compound **7**.

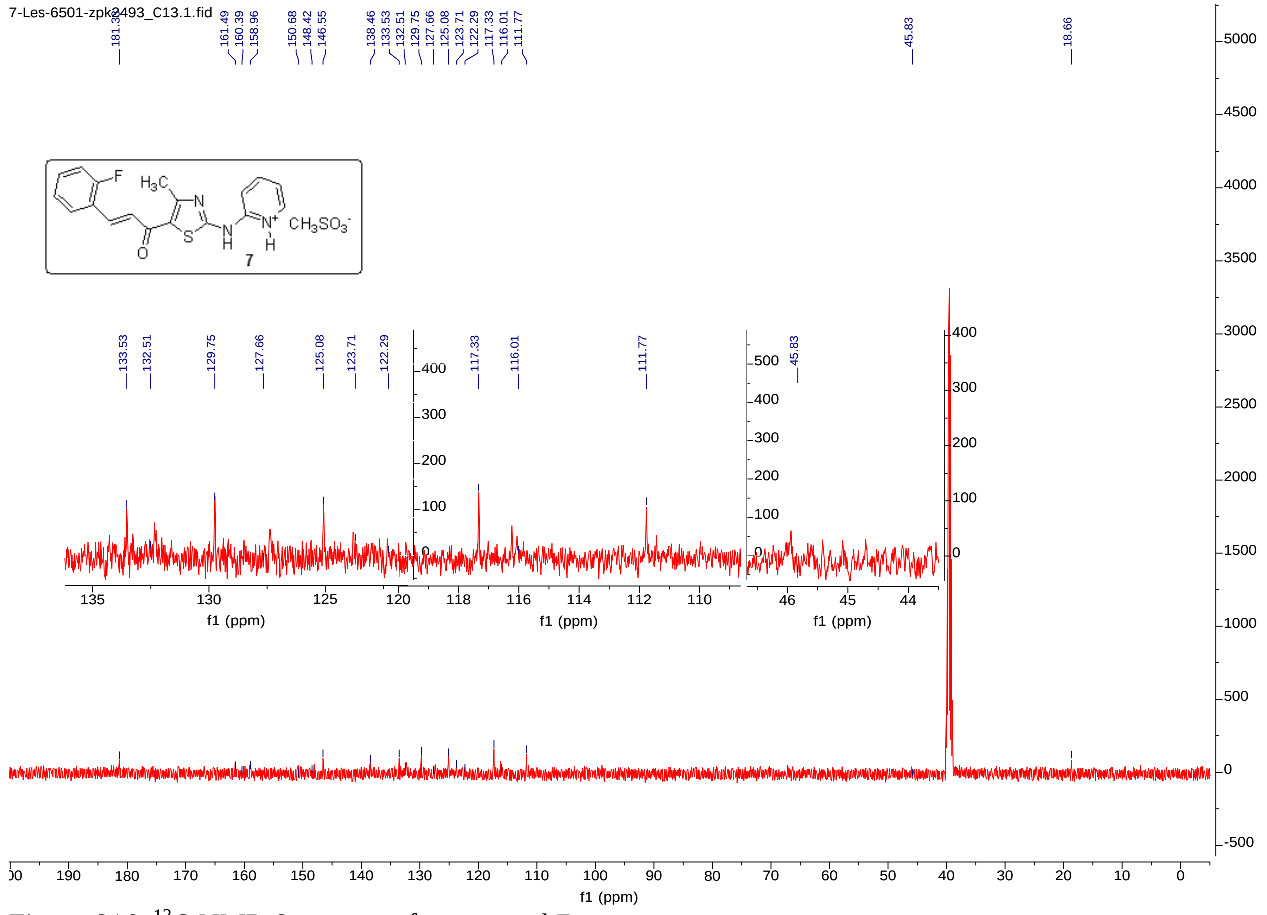

Figure S16. ^{13}C NMR Spectrum of compound **7**.

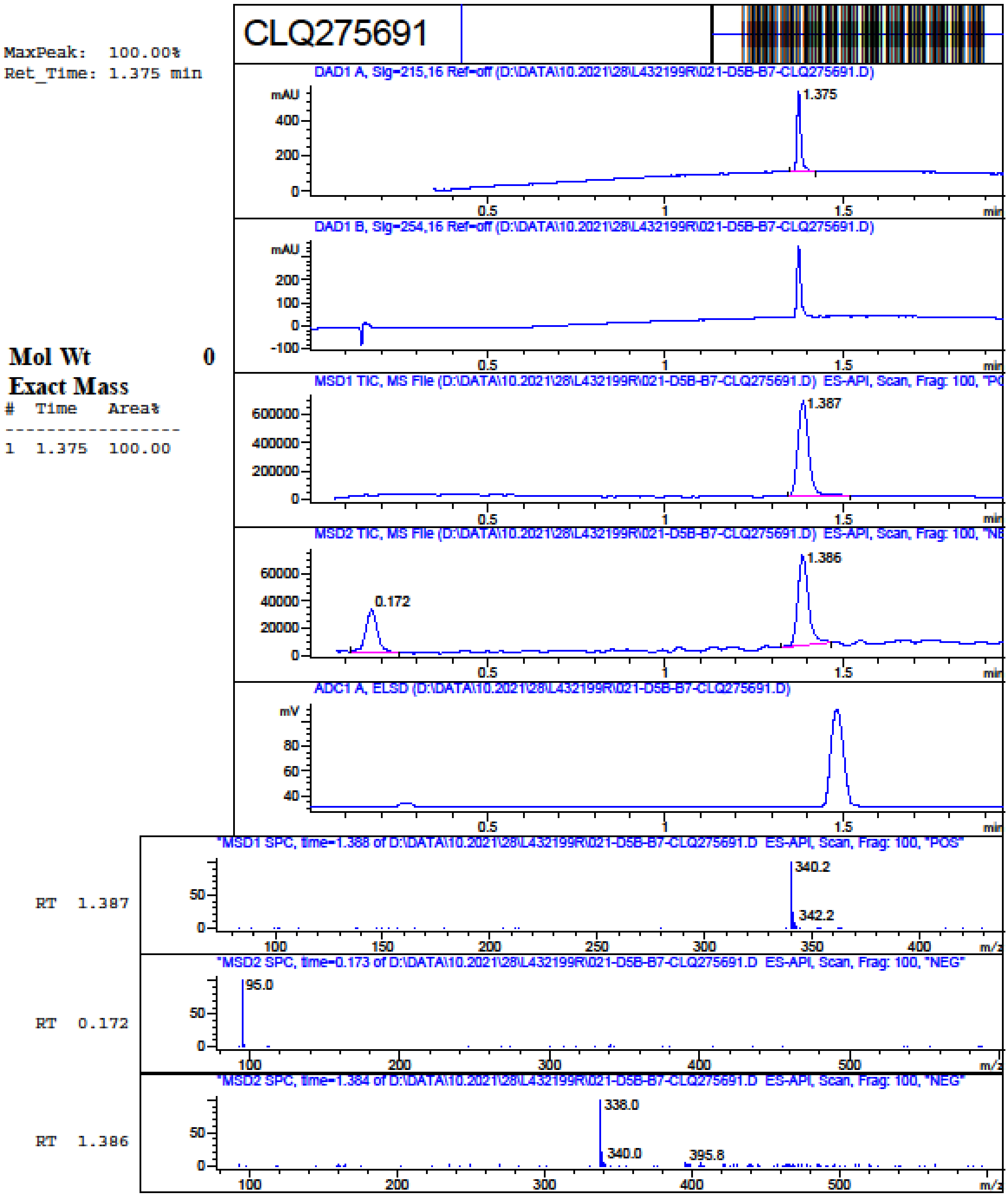

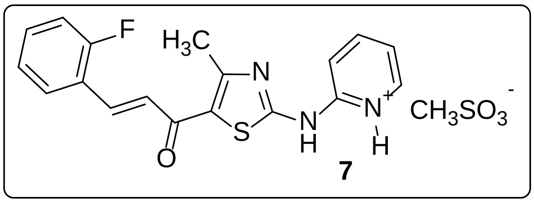

Molecular Weight: 435,49

Figure S17. LCMS Spectrum of compound **7**.

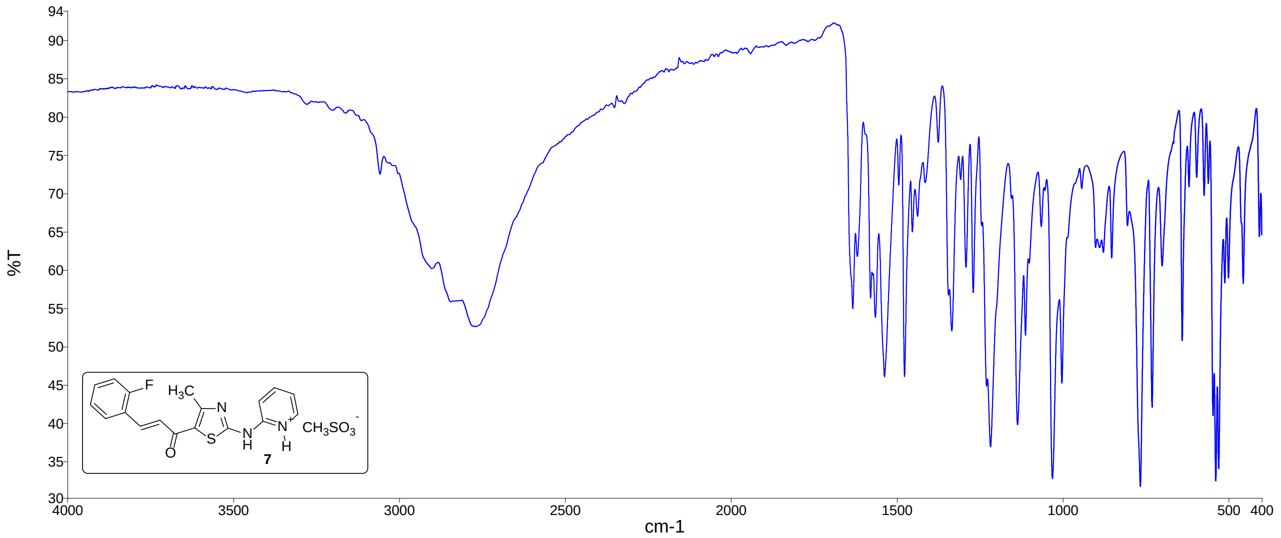

Figure S18. IR Spectrum of compound **7**.

Developmental Therapeutics Program One Dose Mean Graph	NSC: D-832924 / 1	Conc: 1.00E-5 Molar	Test Date: Oct 18, 2021
	Experiment ID: 2110OS01		Report Date: Nov 20, 2021

Panel/Cell Line	Growth Percent
**Leukemia**	
CCRF-CEM	3.92
HL-60(TB)	2.80
K-562	5.79
MOLT-4	11.50
RPMI-8226	-14.16
SR	4.27
**Non-Small Cell Lung Cancer**	
A549/ATCC	1.12
EKVX	20.19
HOP-62	3.55
HOP-92	-14.52
NCI-H226	4.65
NCI-H23	0.66
NCI-H322M	17.54
NCI-H460	-40.69
NCI-H522	-22.73
**Colon Cancer**	
COLO 205	-34.08
HCC-2998	-7.21
HCT-116	1.05
HCT-15	-42.04
HT29	-28.32
KM12	-50.82
SW-620	0.24
**CNS Cancer**	
SF-268	-0.24
SF-295	0.23
SF-539	-27.26
SNB-19	21.08
SNB-75	-3.07
U251	-70.41
**Melanoma**	
LOX IMVI	-76.78
MALME-3M	34.23
M14	0.87
MDA-MB-435	-10.70
SK-MEL-2	-31.22
SK-MEL-28	33.31
SK-MEL-5	7.25
UACC-257	39.01
UACC-62	11.82
**Ovarian Cancer**	
IGROV1	14.01
OVCAR-3	6.50
OVCAR-4	41.12
OVCAR-5	-6.86
OVCAR-8	-0.28
NCI/ADR-RES	10.51
SK-OV-3	5.92
**Renal Cancer**	
786-0	-12.50
A498	-1.49
ACHN	12.13
CAKI-1	2.96
RXF 393	-12.06
SN12C	-14.76
TK-10	28.70
UO-31	-2.98
**Prostate Cancer**	
PC-3	0.81
DU-145	1.39
**Breast Cancer**	
MCF7	-17.27
MDA-MB-231/ATCC	10.52
HS 578T	8.86
BT-549	-9.95
T-47D	13.07
MDA-MB-468	3.80
Mean	-2.78
Delta	74.00
Range	117.90

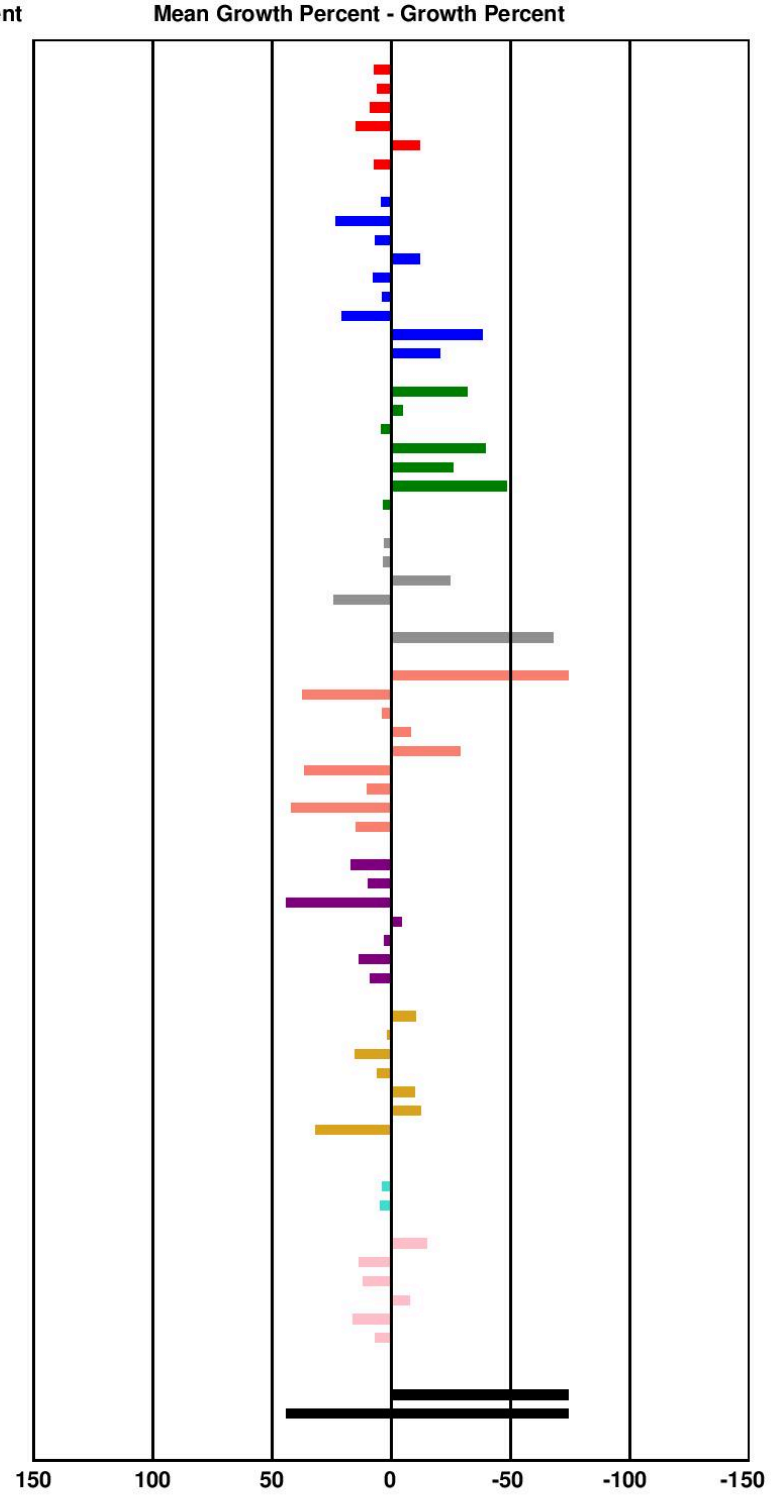

Figure S19. NCI-60 cell lines screening protocol in concentration 10 μM for compound **4**.

National Cancer Institute Developmental Therapeutics Program
In-Vitro Testing Results

NSC : D - 832924 / 1	Experiment ID : 2112NS20	Test Type : 08	Units : Molar
Report Date : February 16, 2022	Test Date : December 06, 2021	QNS :	MC :
COMI : Les-6485	Stain Reagent : SRB Dual-Pass Related	SSPL : 0PZO	

			Log10 Concentration												
	Time		Mean Optical Densities					Percent Growth							
Panel/Cell Line	Zero	Ctrl	-8.0	-7.0	-6.0	-5.0	-4.0	-8.0	-7.0	-6.0	-5.0	-4.0	GI50	TGI	LC50
**Leukemia**															
CCRF-CEM	0.402	2.400	2.354	2.277	2.083	1.703	0.589	98	94	84	65	9	1.86E-5	> 1.00E-4	> 1.00E-4
HL-60(TB)	0.523	2.682	2.534	2.630	2.398	2.088	0.620	93	98	87	72	4	2.14E-5	> 1.00E-4	> 1.00E-4
K-562	0.193	1.983	1.949	2.110	1.824	1.576	0.567	98	107	91	77	21	3.04E-5	> 1.00E-4	> 1.00E-4
MOLT-4	0.524	2.400	2.530	2.615	2.564	2.308	0.641	107	111	109	95	6	3.22E-5	> 1.00E-4	> 1.00E-4
RPMI-8226	0.758	2.884	2.849	2.799	2.131	1.794	1.029	98	96	65	49	13	8.29E-6	> 1.00E-4	> 1.00E-4
SR	0.415	2.158	2.116	2.186	2.042	1.655	0.691	98	102	93	71	16	2.41E-5	> 1.00E-4	> 1.00E-4
**Non-Small Cell Lung Cancer**															
A549/ATCC	0.415	2.461	2.336	2.395	2.317	2.164	1.339	94	97	93	85	45	7.57E-5	> 1.00E-4	> 1.00E-4
EKVX	0.717	1.610	1.532	1.596	1.555	1.316	0.769	91	98	94	67	6	1.90E-5	> 1.00E-4	> 1.00E-4
HOP-62	0.562	1.749	1.680	1.728	1.608	1.409	0.967	94	98	88	71	34	3.75E-5	> 1.00E-4	> 1.00E-4
HOP-92	1.188	1.985	1.885	1.871	1.827	1.616	1.310	87	86	80	54	15	1.25E-5	> 1.00E-4	> 1.00E-4
NCI-H226	0.788	1.414	1.300	1.294	1.179	0.985	0.771	82	81	62	31	-2	2.52E-6	8.59E-5	> 1.00E-4
NCI-H23	0.648	1.881	1.792	1.860	1.688	1.490	0.859	93	98	84	68	17	2.27E-5	> 1.00E-4	> 1.00E-4
NCI-H322M	0.915	2.278	2.204	2.257	2.111	2.012	0.987	95	98	88	80	5	2.54E-5	> 1.00E-4	> 1.00E-4
NCI-H460	0.312	3.082	3.114	3.136	2.948	2.669	0.702	101	102	95	85	14	3.12E-5	> 1.00E-4	> 1.00E-4
NCI-H522	1.137	2.612	2.416	2.421	2.324	2.126	1.377	87	87	80	67	16	2.16E-5	> 1.00E-4	> 1.00E-4
**Colon Cancer**															
COLO 205	0.977	3.027	3.025	3.069	3.048	2.989	2.142	100	102	101	98	57	> 1.00E-4	> 1.00E-4	> 1.00E-4
HCC-2998	0.594	1.849	1.795	1.887	1.821	1.571	0.610	96	103	98	78	1	2.31E-5	> 1.00E-4	> 1.00E-4
HCT-116	0.297	2.588	2.512	2.524	2.386	1.986	0.482	97	97	91	74	8	2.30E-5	> 1.00E-4	> 1.00E-4
HCT-15	0.280	1.707	1.598	1.664	1.626	1.354	0.478	92	97	94	75	14	2.58E-5	> 1.00E-4	> 1.00E-4
HT29	0.343	2.191	2.128	2.280	2.212	2.100	1.012	97	105	101	95	36	5.83E-5	> 1.00E-4	> 1.00E-4
KM12	0.807	3.246	3.350	3.289	3.157	3.034	1.682	104	102	96	91	36	5.56E-5	> 1.00E-4	> 1.00E-4
SW-620	0.252	2.175	2.164	2.156	1.959	1.940	1.112	99	99	89	88	45	7.53E-5	> 1.00E-4	> 1.00E-4
**CNS Cancer**															
SF-268	0.853	2.382	2.313	2.422	2.375	1.926	1.440	95	103	100	70	38	4.31E-5	> 1.00E-4	> 1.00E-4
SF-295	0.671	2.728	2.565	2.677	2.541	2.281	1.138	92	97	91	78	23	3.22E-5	> 1.00E-4	> 1.00E-4
SF-539	0.788	2.320	2.270	2.343	2.156	1.963	1.166	97	101	89	77	25	3.25E-5	> 1.00E-4	> 1.00E-4
SNB-19	0.756	2.517	2.352	2.436	2.309	2.254	1.165	91	95	88	85	23	3.69E-5	> 1.00E-4	> 1.00E-4
SNB-75	0.878	1.673	1.623	1.558	1.477	1.114	0.760	94	85	75	30	-13	3.58E-6	4.88E-5	> 1.00E-4
U251	0.555	2.269	2.217	2.262	2.202	2.044	1.114	97	100	96	87	33	4.78E-5	> 1.00E-4	> 1.00E-4
**Melanoma**															
LOX IMVI	0.405	2.682	2.332	1.673	1.411	1.228	0.565	85	56	44	36	7	3.12E-7	> 1.00E-4	> 1.00E-4
MALME-3M	0.754	1.642	1.630	1.611	1.485	1.339	0.991	99	96	82	66	27	2.54E-5	> 1.00E-4	> 1.00E-4
M14	0.530	2.029	2.012	1.997	1.758	1.470	0.809	99	98	82	63	19	1.94E-5	> 1.00E-4	> 1.00E-4
MDA-MB-435	0.679	2.952	3.018	3.006	2.893	2.492	0.883	103	102	97	80	9	2.63E-5	> 1.00E-4	> 1.00E-4
SK-MEL-2	1.404	2.934	2.849	2.908	2.810	2.637	1.919	94	98	92	81	34	4.49E-5	> 1.00E-4	> 1.00E-4
SK-MEL-28	0.656	2.160	2.247	2.331	2.146	1.895	1.068	106	111	99	82	27	3.87E-5	> 1.00E-4	> 1.00E-4
SK-MEL-5	0.951	3.140	3.129	3.121	2.998	2.502	0.888	99	99	94	71	-7	1.86E-5	8.20E-5	> 1.00E-4
UACC-257	0.750	1.920	1.851	1.922	1.832	1.647	1.114	94	100	92	77	31	3.84E-5	> 1.00E-4	> 1.00E-4
UACC-62	0.944	3.110	3.067	3.080	2.784	2.279	1.060	98	99	85	62	5	1.61E-5	> 1.00E-4	> 1.00E-4
**Ovarian Cancer**															
IGROV1	0.747	2.719	2.714	2.677	2.512	1.830	0.712	100	98	90	55	-5	1.21E-5	8.32E-5	> 1.00E-4
OVCAR-3	0.664	2.182	2.275	2.286	2.100	1.854	0.996	106	107	95	78	22	3.17E-5	> 1.00E-4	> 1.00E-4
OVCAR-4	0.769	1.996	1.954	2.045	2.088	1.914	1.266	97	104	107	93	41	6.61E-5	> 1.00E-4	> 1.00E-4
OVCAR-5	0.641	1.880	1.918	1.987	1.893	1.609	1.209	103	109	101	78	46	7.44E-5	> 1.00E-4	> 1.00E-4
OVCAR-8	0.934	3.239	3.219	3.258	3.197	2.945	1.768	99	101	98	87	36	5.36E-5	> 1.00E-4	> 1.00E-4
NCI/ADR-RES	0.536	1.760	1.705	1.770	1.651	1.515	0.557	95	101	91	80	2	2.41E-5	> 1.00E-4	> 1.00E-4
SK-OV-3	0.859	1.815	1.745	1.781	1.723	1.478	1.138	93	96	90	65	29	2.60E-5	> 1.00E-4	> 1.00E-4
**Renal Cancer**															
786-0	0.525	2.259	2.111	2.078	2.004	1.675	0.959	91	90	85	66	25	2.48E-5	> 1.00E-4	> 1.00E-4
A498	1.190	2.051	2.020	2.088	2.045	1.905	1.566	96	104	99	83	44	6.92E-5	> 1.00E-4	> 1.00E-4
ACHN	0.484	1.964	1.890	2.020	1.834	1.505	1.079	95	104	91	69	40	4.56E-5	> 1.00E-4	> 1.00E-4
CAKI-1	0.646	2.291	2.198	2.171	1.964	1.508	0.845	94	93	80	52	12	1.15E-5	> 1.00E-4	> 1.00E-4
RXF 393	1.166	1.711	1.669	1.677	1.578	1.240	0.897	92	94	76	14	-23	2.59E-6	2.35E-5	> 1.00E-4
SN12C	0.593	2.419	2.362	2.469	2.223	1.878	1.046	97	103	89	70	25	2.80E-5	> 1.00E-4	> 1.00E-4
TK-10	1.365	2.264	2.198	2.216	2.329	2.515	1.750	93	95	107	128	43	8.23E-5	> 1.00E-4	> 1.00E-4
UO-31	0.834	2.866	2.682	2.439	2.041	1.662	1.108	91	79	59	41	13	3.19E-6	> 1.00E-4	> 1.00E-4
**Prostate Cancer**															
PC-3	0.617	2.348	2.217	2.260	2.123	1.837	0.897	92	95	87	70	16	2.38E-5	> 1.00E-4	> 1.00E-4
DU-145	0.467	1.812	1.763	1.843	1.865	1.682	1.026	96	102	104	90	42	6.72E-5	> 1.00E-4	> 1.00E-4
**Breast Cancer**															
MCF7	0.316	1.796	1.684	1.810	1.581	1.200	0.429	92	101	86	60	8	1.54E-5	> 1.00E-4	> 1.00E-4
MDA-MB-231/ATCC	0.619	1.791	1.814	1.785	1.590	1.312	0.940	102	99	83	59	27	1.93E-5	> 1.00E-4	> 1.00E-4
HS 578T	1.452	2.828	2.710	2.746	2.719	2.287	1.431	91	94	92	61	-1	1.49E-5	9.48E-5	> 1.00E-4
BT-549	1.346	2.501	2.518	2.475	2.398	2.054	1.266	101	98	91	61	-6	1.47E-5	8.15E-5	> 1.00E-4
T-47D	0.902	2.418	2.208	2.168	2.232	1.863	1.069	86	84	88	63	11	1.80E-5	> 1.00E-4	> 1.00E-4
MDA-MB-468	0.890	1.616	1.534	1.524	1.455	1.134	0.727	89	87	78	34	-18	4.24E-6	4.43E-5	> 1.00E-4

Figure S20a. NCI-60 cell lines screening protocol concentrations ranging from 10^{-4} to 10^{-8} M for compound **4**.

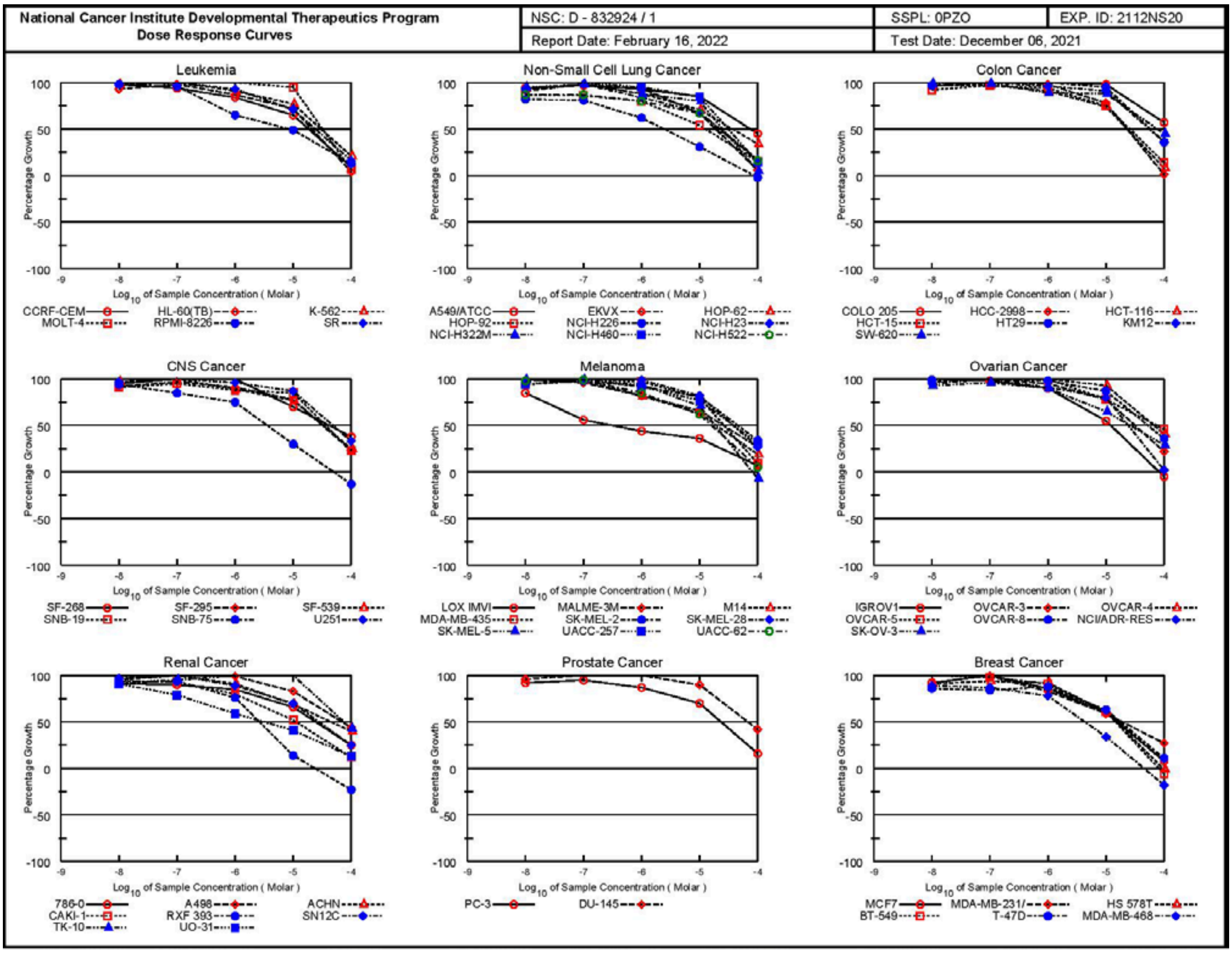

Figure S20b. NCI-60 cell lines screening protocol concentrations ranging from 10^{-4} to 10^{-8} M for compound **4**.

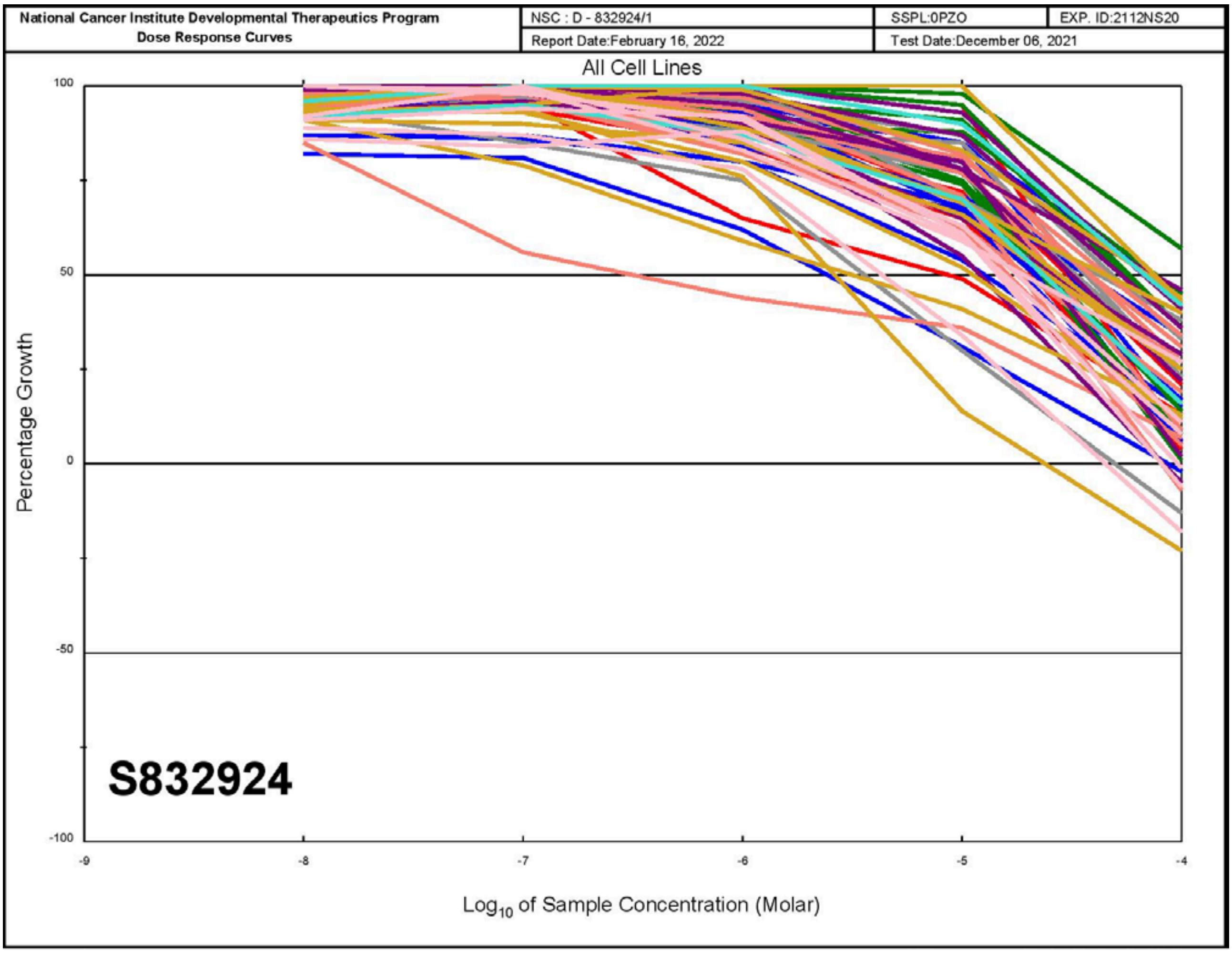

Figure S20c. NCI-60 cell lines screening protocol concentrations ranging from 10^{-4} to 10^{-8} M for compound **4**.

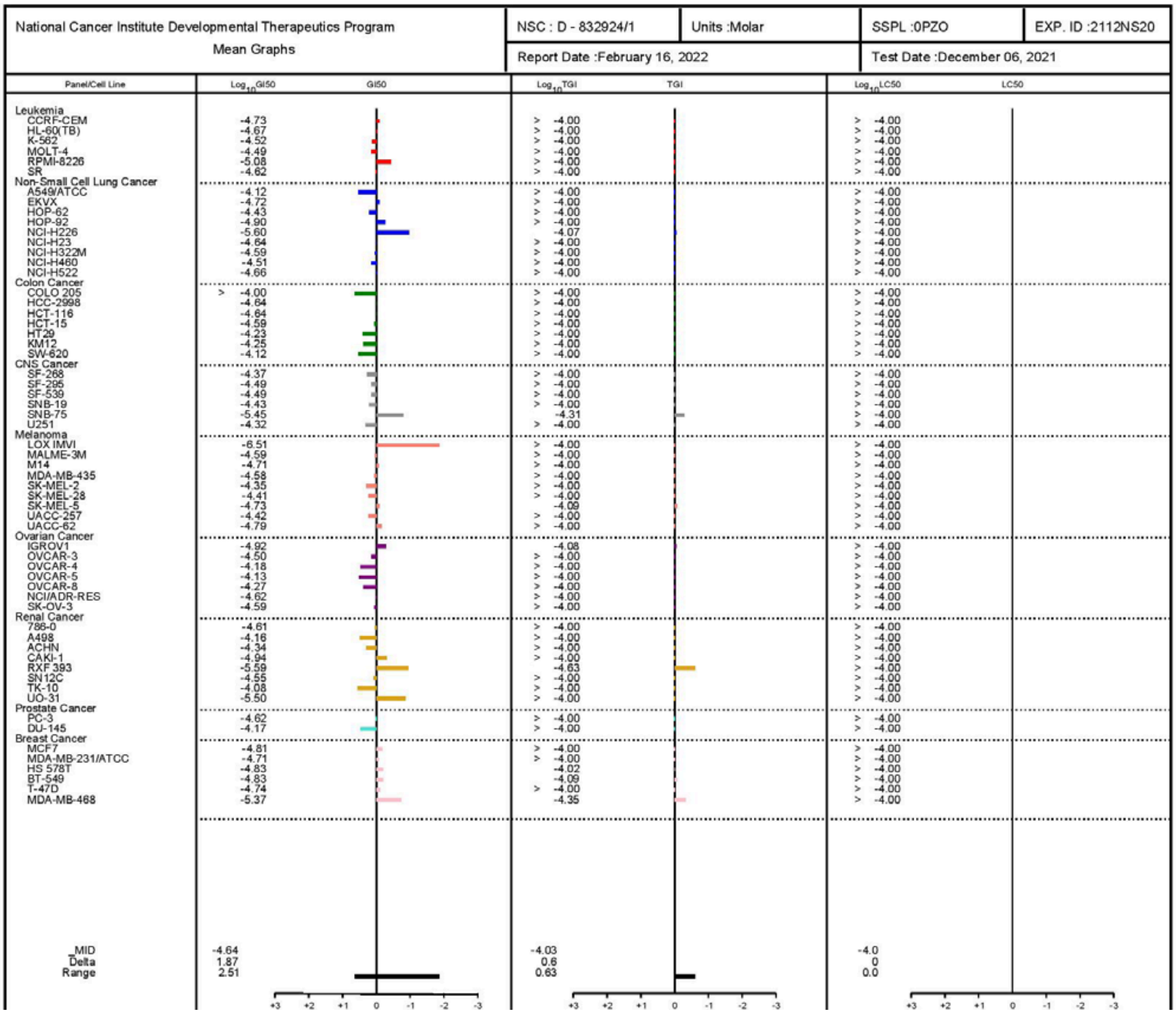

Figure S20d. NCI-60 cell lines screening protocol concentrations ranging from 10^{-4} to 10^{-8} M for compound **4**.

Developmental Therapeutics Program One Dose Mean Graph	NSC: D-836191 / 1	Conc: 1.00E-5 Molar	Test Date: Mar 14, 2022
	Experiment ID: 2203OS89		Report Date: Apr 06, 2022

Panel/Cell Line	Growth Percent
Leukemia	
CCRF-CEM	19.12
HL-60(TB)	4.96
K-562	15.13
MOLT-4	25.36
RPMI-8226	30.40
SR	12.44
Non-Small Cell Lung Cancer	
A549/ATCC	59.19
EKVX	72.39
HOP-62	70.61
HOP-92	62.45
NCI-H226	78.91
NCI-H23	67.65
NCI-H322M	89.07
NCI-H460	28.04
NCI-H522	-18.90
Colon Cancer	
COLO 205	85.96
HCC-2998	95.75
HCT-116	34.32
HCT-15	20.77
HT29	30.43
KM12	23.37
SW-620	32.14
CNS Cancer	
SF-268	78.24
SF-295	39.64
SF-539	32.42
SNB-19	56.09
SNB-75	62.33
U251	51.05
Melanoma	
LOX IMVI	31.49
MALME-3M	63.77
M14	52.61
MDA-MB-435	-17.91
SK-MEL-2	62.07
SK-MEL-28	65.95
SK-MEL-5	48.67
UACC-257	83.15
UACC-62	29.45
Ovarian Cancer	
IGROV1	64.19
OVCAR-3	24.77
OVCAR-4	74.78
OVCAR-5	82.77
OVCAR-8	67.97
NCI/ADR-RES	20.95
SK-OV-3	98.30
Renal Cancer	
786-0	57.60
A498	100.96
ACHN	56.53
CAKI-1	61.81
RXF 393	87.47
SN12C	57.90
TK-10	118.83
UO-31	47.96
Prostate Cancer	
PC-3	58.54
DU-145	67.35
Breast Cancer	
MCF7	20.14
MDA-MB-231/ATCC	47.50
HS 578T	87.63
BT-549	36.14
T-47D	33.49
MDA-MB-468	-13.74
Mean	50.64
Delta	69.54
Range	137.73

Mean Growth Percent - Growth Percent

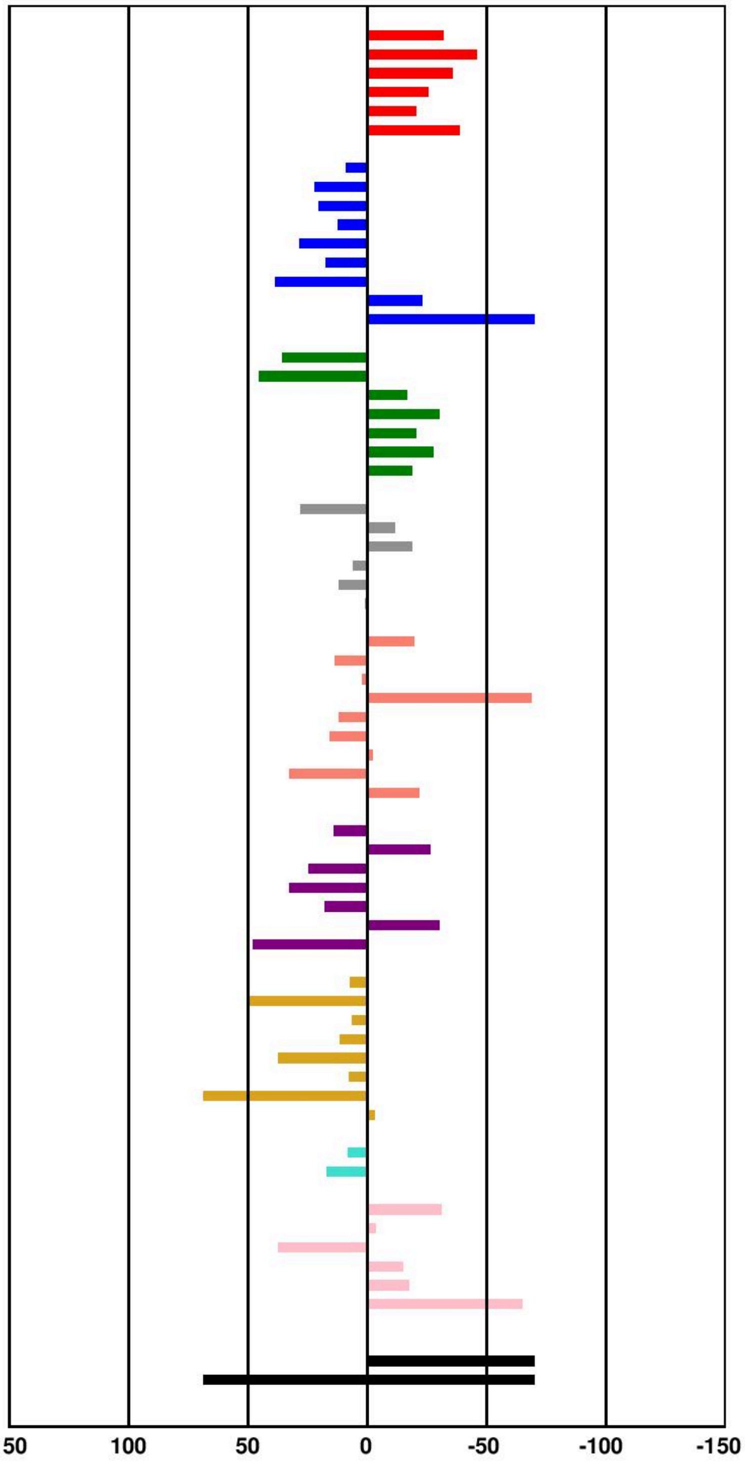

Figure S21. NCI-60 cell lines screening protocol in concentration 10 μM for compound **5**.

Developmental Therapeutics Program

One Dose Mean Graph

**NSC:** D-833525 / 1	**Conc:** 1.00E-5 Molar	**Test Date:** Nov 29, 2021
**Experiment ID:** 2111OS18		**Report Date:** Jan 14, 2022

Panel/Cell Line	Growth Percent
Leukemia	
CCRF-CEM	44.19
HL-60(TB)	45.37
K-562	15.62
MOLT-4	28.85
RPMI-8226	43.97
SR	12.53
Non-Small Cell Lung Cancer	
A549/ATCC	33.69
EKVX	44.89
HOP-62	87.59
HOP-92	44.29
NCI-H226	53.38
NCI-H23	79.30
NCI-H322M	86.12
NCI-H460	31.44
NCI-H522	45.83
Colon Cancer	
COLO 205	46.08
HCC-2998	95.32
HCT-116	31.18
HCT-15	23.24
HT29	40.70
KM12	49.64
SW-620	80.29
CNS Cancer	
SF-268	73.35
SF-295	38.77
SF-539	58.66
SNB-19	60.62
SNB-75	83.76
U251	44.54
Melanoma	
MALME-3M	64.03
M14	58.91
MDA-MB-435	35.91
SK-MEL-2	76.04
SK-MEL-28	62.69
SK-MEL-5	17.75
UACC-257	60.40
UACC-62	44.56
Ovarian Cancer	
IGROV1	61.61
OVCAR-3	55.81
OVCAR-4	41.79
OVCAR-5	82.81
OVCAR-8	60.06
NCI/ADR-RES	50.59
SK-OV-3	85.49
Renal Cancer	
786-0	40.03
A498	84.16
ACHN	61.67
CAKI-1	51.74
RXF 393	45.99
SN12C	47.48
TK-10	52.55
UO-31	43.91
Prostate Cancer	
PC-3	22.55
DU-145	54.79
Breast Cancer	
MCF7	45.80
MDA-MB-231/ATCC	44.72
HS 578T	78.64
BT-549	30.31
T-47D	14.27
MDA-MB-468	37.20
Mean	51.48
Delta	38.95
Range	82.79

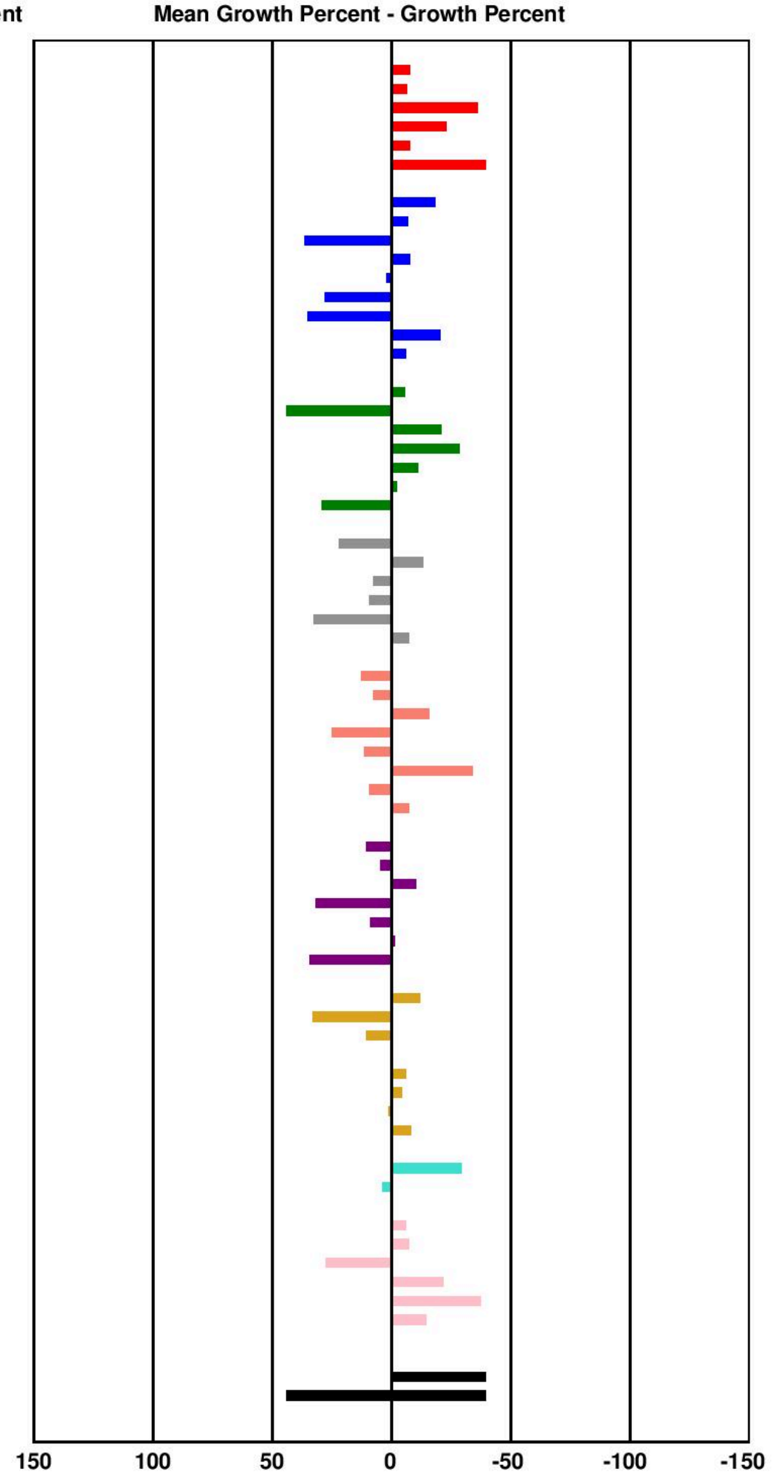

Figure S22. NCI-60 cell lines screening protocol in concentration 10 μM for compound **6**.
